# Electroferrofluids with nonequilibrium voltage-controlled magnetism, diffuse interfaces, and patterns

**DOI:** 10.1126/sciadv.abi8990

**Published:** 2021-12-22

**Authors:** Tomy Cherian, Fereshteh Sohrabi, Carlo Rigoni, Olli Ikkala, Jaakko V. I. Timonen

**Affiliations:** Department of Applied Physics, Aalto University School of Science, Puumiehenkuja 2, Espoo 02150, Finland.

## Abstract

It has been recognized that driving matter to nonequilibrium states can lead to emergent behaviors and functionalities. Here, we show that uniform colloidal dispersions can be driven into dissipative nonuniform states with emerging behaviors. We experimentally demonstrate this with electrically driven weakly charged superparamagnetic iron oxide nanoparticles in a nonpolar solvent. The driving leads to formation of nonequilibrium concentration gradients that further translate to nonequilibrium magnetism, including voltage-controlled magnetization and susceptibility. The concentration gradients also serve as diffuse interfaces that respond to external magnetic fields, leading to novel dissipative patterns. We identify the closest nondissipative analogs, discuss the differences, and highlight the ability to directly quantify the dissipation and link it to the pattern formation. Beyond voltage-controlled magnetism, we foresee that the concept can be generalized to other functional colloids to create, e.g., optical, electrical, catalytic, and mechanical responses that are not possible in thermodynamic equilibrium.

## Teaser

Electrically driven dissipative gradients of superparamagnetic nanoparticles allow voltage-controlled magnetism and patterns.

## INTRODUCTION

**Fig. 1.**
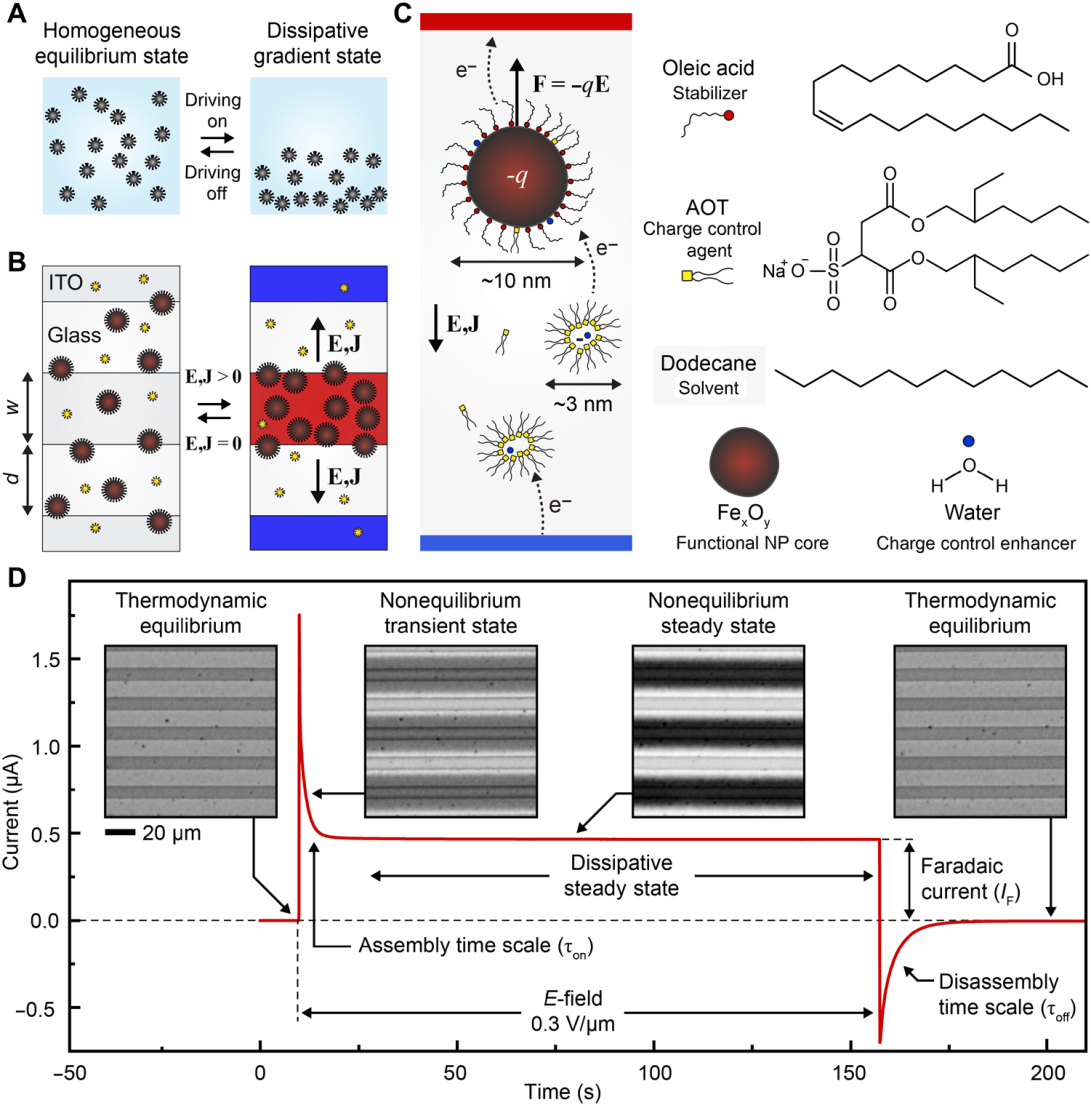
Nonequilibrium steady states in electrically driven colloidal NP dispersions. (**A**) A conceptual scheme of driving a homogeneous colloidal dispersion into a nonhomogeneous dissipative state. (**B**) A scheme of a proposed realization of the concept in (A) by driving charged NPs into gradients with an electric field (**E**) and in presence of an electric current (**J**). (**C**) A scheme showing the experimental realization using oleic acid–coated iron oxide NPs charged using AOT reverse micelles in a nonpolar solvent (electroferrofluid) and the expected charge transfer processes. (**D**) Electric current as a function of time across the electroferrofluid (6% iron oxide NPs and 150 mM AOT in dodecane) in a microelectrode cell (*U* = 3 V, *E* = 0.3 V/μm). Insets show transmitted light microscopy images of the cell at different times (movie S1).

Dissipative structures can exhibit functionalities unparalleled with equilibrium systems (*1*–*5*). While important macroscopic model systems have been developed (*6*, *7*), realizations of microscopic generic platforms with nonequilibrium responses have been limited. Only very recently, synthetic molecular systems driven by chemical fuels (*8*, *9*) and colloidal systems of nanoparticles (NPs) driven by ultraviolet light (*10*), oscillating pH (*11*), chemical fuels (*12*, *13*), or electric fields (*14*) have illustrated useful nonequilibrium responses. However, systematic platforms to drive nonequilibrium steady states for emergent functionalities are still scarce. Here, we propose that driving homogeneous NP dispersions into concentration gradients (Fig. 1A) with sustained energy dissipation induces novel functional responses. This is shown here for the case of magnetic colloids leading to emergence of dissipative voltage–controlled magnetism and patterns, with a foreseeable generalization to, e.g., optical, mechanical, and catalytic responses using other functional NPs.

## RESULTS AND DISCUSSION

### Demonstration of electrically driven dissipative NP gradients

Our system is based on electrical driving of weakly charged superparamagnetic iron oxide NPs stabilized with oleic acid in dodecane (Fig. 1, B and C, and see text S1 and fig. S1 for characterization). For electrical responsivity, we add bis(2-ethylhexyl) sulfosuccinate sodium salt [aerosol OT (AOT)] as a charge control agent that forms charge-carrying reverse micelles and charges the NPs (Fig. 1C) (*15*–*17*). We incubate the mixture in a humidity chamber to control the water content, which enhances conductivity in the AOT/dodecane system (*18*), resulting in a complex electrically active mixture (see text S2 for characterization).

The electrically active mixture is driven out of the homogeneous thermodynamic equilibrium state by applying an electric field (*E*-field) using microelectrode cells with planar interdigitated indium tin oxide (ITO) electrodes (width *w* = 10 μm, spacing *d* = 10 μm, and cell height *h* = 4 μm; see fig. S2). The *E*-field is approximately uniform and in-plane between the microelectrodes with strength *E* = *U*/*d* (fig. S2). Application of the electric field results in a brief peak in the electric current followed by settling down to a steady-state value *I*_F_ (Fig. 1D). This is accompanied by migration of the NPs to the vicinity of the positive electrodes to form concentration gradients, as seen in the optical micrographs, suggesting that the NPs are negatively charged (movie S1). Once the field is switched off, the current relaxes to zero, and the NPs redisperse back to the homogeneous equilibrium state. The nonequilibrium states can be sustained almost indefinitely.

### Mechanisms behind the gradient formation

**Fig. 2.**
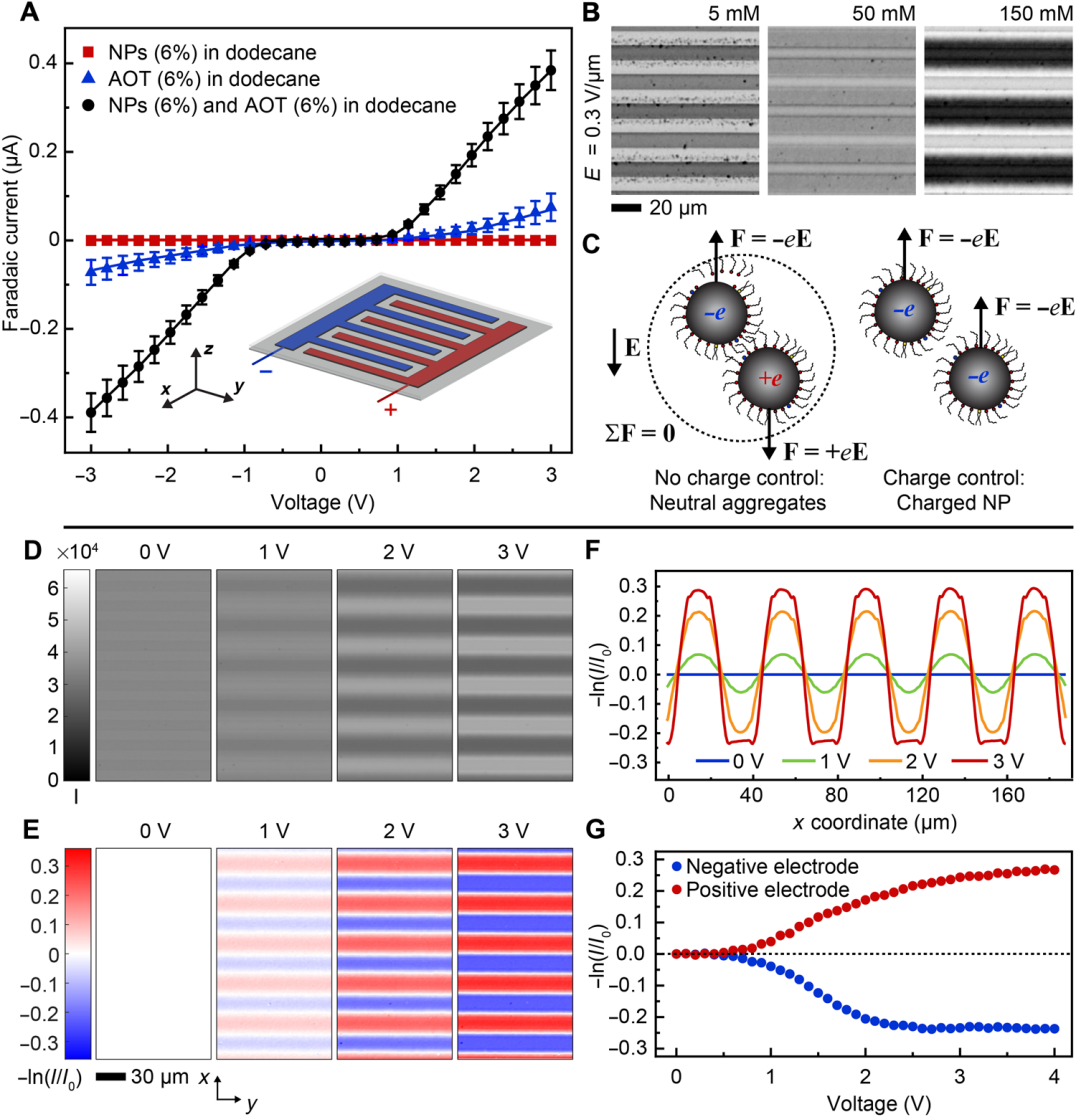
Charge-carrying superparamagnetic NPs enable electrically controlled magnetism in electroferrofluids. (**A**) *I*-*V* curves of iron oxide NPs in dodecane, AOT in dodecane, and the NPs + AOT in dodecane. All percentages indicate volume fractions (6% of AOT corresponds approximately to 150 mM). (**B**) Optical microscopy images showing the steady state of the NPs at different AOT concentrations at *U* = 3 V. (**C**) A scheme of the NP charging states and interactions at low (left) and high (right) AOT concentrations. (**D**) Unprocessed optical microscopy images showing the steady-state NP gradients at different voltages in dodecane with 150 mM AOT. (**E**) Colormaps showing –ln(*I*/*I*_0_) calculated from the images shown in (D). (**F**) Averaged one-dimensional plots of –ln(*I*/*I*_0_) as a function of *x* coordinate obtained from the colormaps in (E). (**G**) –ln(*I*/*I*_0_) as a function of applied voltage on top of the negative and positive electrodes.

Control experiments performed in polar media (water) with highly charged citrate-stabilized iron oxide NPs show no gradient formation in the microelectrode cells under similar electrical driving. Instead, electrodeposition of the highly charged NPs on the ITO electrodes is observed in the aqueous environment (fig. S3 and text S3). This suggests the importance of using weakly charged NPs, enabled by the nonpolar environment together with the AOT as the charge control agent. The AOT concentration was observed to be critical for both the electrical conductivity (Fig. 2A) and the NP behavior (Fig. 2B). At small AOT concentrations, the colloidal dispersion neither conducts electricity nor dissipates energy (Fig. 2A). Furthermore, the NPs were observed to aggregate irreversibly when the electric field was applied (Fig. 2B and movie S2). The poor conductivity is in agreement with the lack of charge carriers (lack of both charged reverse micelles and charged NPs), and the NP aggregation is attributed to the generation of oppositely charged NPs near the positive and negative electrodes and their Coulombic attraction (Fig. 2C). The Coulombic interaction energy *E*_c_ *= e*^2^/(*4*πε*_r_*ε*_0_d*) is especially large in the nonpolar environment due to the low relative permittivity ε_r_ ≈ 2, surpassing the thermal energy even at distances larger than the NP diameter (*E*_c_ *~ -3k*_B_*T* at *d* = 10 nm), driving the aggregation. In contrast, for AOT concentrations at and above 50 mM, the NP aggregation was suppressed, and the gradients appeared (Fig. 2B). The gradients intensified when the AOT concentration was increased (fig. S4).

The higher conductivity of the electroferrofluid containing 150 mM of AOT compared to the plain 150 mM AOT solution in dodecane indicates strong interaction between the AOT and the NPs, leading to notable charging of the NPs that act as additional charge carriers. The nonlinearity of the IV curves of both the 150 mM AOT in dodecane and the electroferrofluid and, especially, the poorly conductive regime below 1 V (Fig. 2A) are likely indications of an electron tunneling process from the electrode into the colloidal dispersion. The initial nonlinearity is followed by ohmic regime at higher voltages that has been observed earlier in AOT solutions (*16*).

### Voltage-controlled magnetism

The NP concentration gradients translate via the superparamagnetism of the NPs to electrically controlled nonequilibrium magnetic responses. The magnetic susceptibility χ and the saturation magnetization *M*_S_ of an NP dispersion are, in the first approximation, linearly proportional to the NP concentration *c*. Using the Beer-Lambert law (text S4), the unprocessed transmitted light images of the steady-state gradients (Fig. 2D, with spatially varying intensity *I*) can be converted to maps of –ln(*I*/*I*_0_) that is a proxy for the normalized change in the NP concentration and, hence, for the change in the magnetic responsiveness (Fig. 2E and fig. S5).

The magnetic responsiveness increases near the positive electrode and decreases near the negative electrode with increasing voltage (Fig. 2E). The responsiveness varies spatially smoothly, approximately sinusoidally, at small driving voltages (*U* = 1 V and *U* = 2 V; Fig. 2F). At higher fields, the smooth perturbations are not observed because of the complete depletion of the NPs from the vicinity of the negative electrodes, leading to flattening of the responsiveness function –ln(*I*/*I*_0_) (*U* = 3 V; Fig. 2F). The magnetic responsiveness contrast increases nonlinearly with the applied voltage (Fig. 2G). The initial nonlinearity can be understood to be linked to the similar behavior in the driving current (Fig. 2A), suggesting the importance of the current and, hence, energy dissipation. The saturation at higher fields is associated to the nearly complete depletion and saturation of the areas near the negative and positive electrodes, respectively (Fig. 2G). The maximum achievable magnetic contrast increases with the increasing NP concentration, as expected (fig. S6).

### Instability of the NP gradients in magnetic field and formation of dissipative patterns

**Fig. 3.**
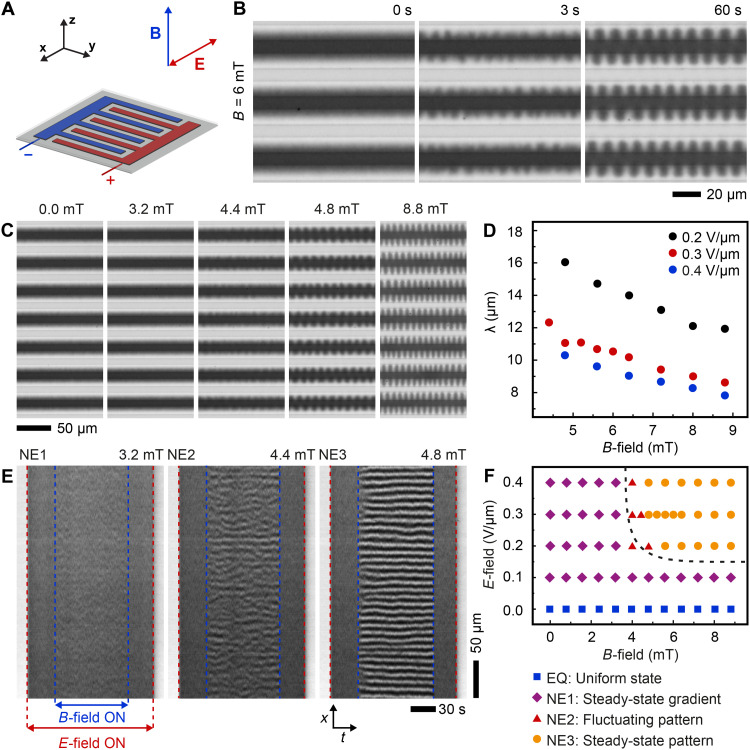
Response of the electroferrofluid (6% iron oxide NPs and 150 mM AOT) to a uniform perpendicular magnetic field *B*$\vec{z}$. (**A**) A scheme of the microelectrode cell with field directions indicated. (**B**) Time series of microscopy images showing the response of an NP gradient (*E =* 0.3 V/μm) to a magnetic field (*B* = 6 mT; movie S3). (**C**) Steady-state microscopy images in a constant electric field (*E* = 0.3 V/μm) and varying magnetic field (*B* = 0 to 8.8 mT; movie S4). (**D**) Average pattern periodicity (λ) as a function of *E* and *B*. (**E**) Kymographs obtained at three different magnetic field strengths near the threshold at a fixed *E* = 0.3 V/μm. (**F**) Phase diagram showing the four states of the electroferrofluid as a function of in-plane *E*-field and perpendicular *B*-field based on steady-state snapshots taken 1 min after turning *B*-field on.

**Fig. 4.**
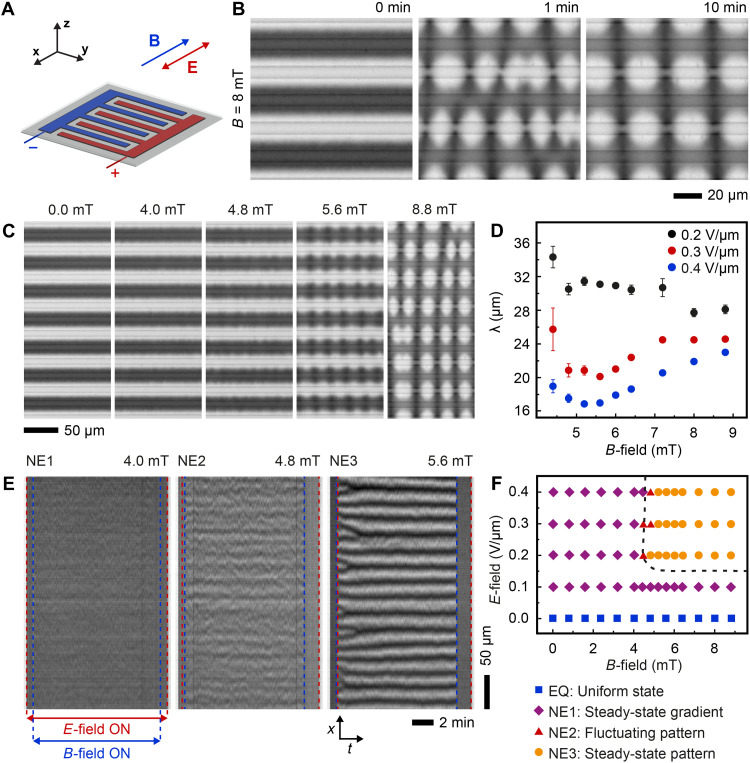
Response of the electroferrofluid (6% iron oxide NPs and 150 mM AOT) to a uniform parallel magnetic field *B*$\vec{x}$. (**A**) A scheme of the microelectrode cell with field directions indicated. (**B**) Time series of microscopy images showing the response of an NP gradient (*E =* 0.3 V/μm) to a magnetic field (*B* = 8 mT; movie S5). (**C**) Steady-state microscopy images in a constant electric field (*E* = 0.3 V/μm) and varying magnetic field (*B* = 0 mT to 8.8 mT; movie S6). (**D**) Average pattern periodicity (λ) as a function of *E* and *B*. (**E**) Kymographs obtained at three different magnetic field strengths near the threshold at a fixed *E* = 0.3 V/μm. (**F**) Phase diagram showing the four states of the electroferrofluid as a function of in-plane *E*-field and parallel *B*-field based on steady-state snapshots taken 1 min after turning *B*-field on.

Ferrofluids enable several functionalities and patterns in magnetic fields (*19*) that, in the electroferrofluids, become also voltage controlled. Here, we consider the response of the electroferrofluid in the nonequilibrium gradient state to a uniform magnetic field *B* applied either perpendicular to the microelectrode cell (Fig. 3 and fig. S7B) or parallel to it along the x direction (Fig. 4 and fig. S7A). In the perpendicular case (Fig. 3A), the steady-state NP gradients become unstable above a threshold *B*_c_ of few mT (Fig. 3B). The instability is seen as deformations in the NP gradient that, after a few seconds, develop to a well-defined quasi–one-dimensional steady-state pattern (Fig. 3B and movie S3). The wavelength of the pattern λ is close to the spacing between electrodes and decreases with the increasing magnetic field (*B*-field) (Fig. 3, C and D, and movie S4). Similarly, increasing *E*-field (and thus susceptibility and magnetization contrast; Fig. 2) leads to decrease in λ (Fig. 3D and fig. S8). While λ is almost independent of the NP concentration (fig. S9A), the threshold magnetic field for the onset of the pattern formation decreases from ca. 22 mT at 0.3% iron oxide content to 8 mT at and above 2.6% (fig. S9B). This is qualitatively expected since the magnetic forces driving the instability increase with the increasing NP concentration.

Kymographic analysis shows that the patterns exhibit fluctuations (Fig. 3E). In particular, near the threshold, i.e., the transition from the steady gradient state (3.2 mT; Fig. 3E) to the steady pattern (4.8 mT; Fig. 3E), there exists an intermediate fluctuating state wherein the pattern appears and disappears randomly in different locations (4.4 mT; Fig. 3E). Thus, the behavior under the perpendicular *B-*field and in-plane electric field forms a phase diagram with one homogeneous equilibrium state and three nonequilibrium states (NE1 to NE3): stable gradient, steady pattern, and a narrow segment corresponding to the fluctuating pattern (Fig. 3F).

The behavior of the electroferrofluid in a *B*-field applied parallel to the microelectrode cell and perpendicular to the microelectrodes (Fig. 4A) resembles qualitatively the behavior in the perpendicular case (Fig. 3). The gradient state still becomes unstable above a threshold field *B*_c_, but the appearance of the patterns is different, and the pattern formation time scales are longer (Fig. 4B and movie S5). The pattern periodicity λ depends on *B* (Fig. 4C and movie S6) but is nonmonotonic (Fig. 4D and fig. S10). We attribute the nonmonotonic behavior to the larger amplitude of the pattern, leading to strong interactions of the patterns between the neighboring positive electrodes (Fig. 4C) that are weak in the perpendicular case (Fig. 3C). Similar to the perpendicular case, there is one equilibrium state and three nonequilibrium states, including the state of the fluctuating pattern (Fig. 4, E and F).

### Comparison to the classic equilibrium ferrofluid patterns

**Fig. 5.**
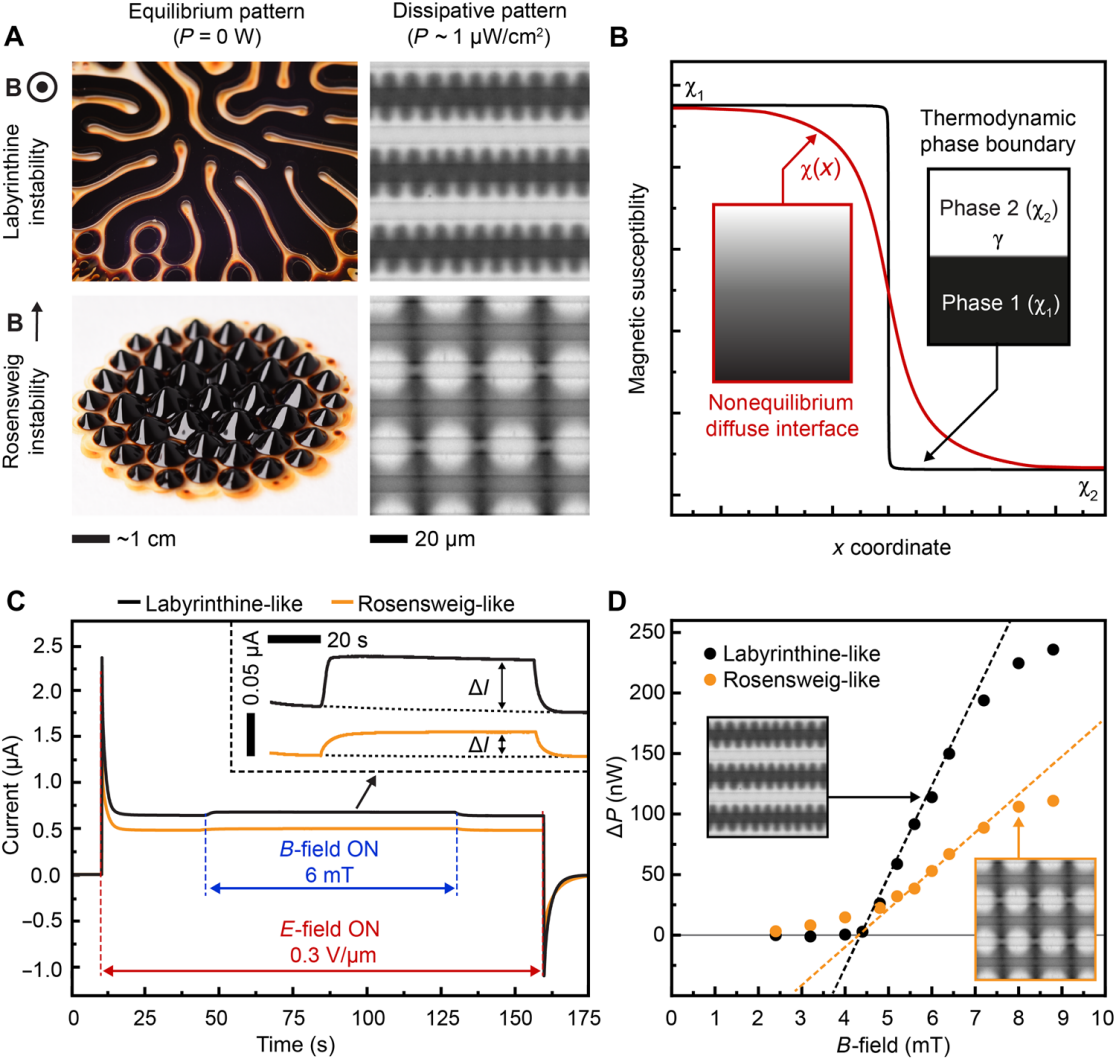
Comparison to classic equilibrium patterns and quantification of dissipation. (**A**) Images of the classic equilibrium Rosensweig and labyrinthine patterns and their dissipative analogs in electroferrofluids. (**B**) A schematic plot of the magnetic susceptibility near an equilibrium thermodynamic interface and a dissipative diffuse interface. (**C**) Electric current passing through the electroferrofluid as a function of time during the formation of the Rosensweig-like and labyrinthine-like patterns at *B* = 6 mT and *E* = 0.3 V/μm. **(D**) Change in dissipated power as a function of magnetic field strength during the formation of the steady-state labyrinthine-like and Rosensweig-like patterns at *E* = 0.3 V/μm.

It is interesting to compare the magnetic response and the patterns of the dissipative electroferrofluid (Figs. 3 and 4) to the classic equilibrium ferrofluid patterns. The latter exhibit two main patterns (*20*): the Rosensweig pattern (*21*, *22*) and the labyrinthine pattern (*23*–*25*) (Fig. 5A). These are caused by a combination of magnetostatic, interfacial, and gravitational forces and are driven by energy minimization. Analogously, the dissipative electroferrofluid patterns can be classified as labyrinthine-like (Fig. 3) and Rosensweig-like patterns (Fig. 4) based on the orientation of the *B*-field with respect to the diffuse interface. However, there are several major differences that are pointed out below.

First, the interfacial tension plays a critical role in the classic patterns and their periodicities. For example, in the classic Rosensweig instability, the pattern wavelength λ is solely determined by the interfacial tension and the gravitational force. However, in electroferrofluids, it is challenging even to define the interfacial tension for the diffuse interfaces (Fig. 5B). Moreover, the gravitational force is negligible at the microelectrode length scales. These major differences suggest that the application of the models developed to describe the classical patterns is not straightforward in the dissipative electroferrofluids. This shows particularly well, for example, in the nonmonotonic dependency of λ with *B* and *E* in the Rosensweig-like configuration (Fig. 4D) with no equivalent behavior seen in the classic systems.

Second, the appearance of the patterns is different compared to the equilibrium patterns (Fig. 5A). This is caused by the diffuse nature of the interface and the confinement in the periodic microelectrode cells. The effect of the confinement can be seen, for example, in the labyrinthine-like patterns, where the spatial size of the pattern on the positive electrode is restricted by the adjacent negative electrodes, leading to the comb-like patterns that are not typically seen in the classic labyrinthine instability (Figs. 3C and 5A). The effect of the confinement can be seen also in the Rosensweig-like patterns where the coupling and alignment of the patterns between the neighboring positive electrodes is evident but which has no equivalent in the classic equilibrium Rosensweig pattern with one layer of magnetic fluid only (Figs. 4C and 5A).

Third, only the electroferrofluids exhibit fluctuating patterns near the threshold (Fig. 3E and Fig. 4E). The origin of the fluctuating pattern is likely in the large NP concentration fluctuations that are present already in the steady-state gradients (see, e.g., movie S1). Because of the large NP concentration fluctuations, and thus fluctuations in the magnetization, the threshold magnetic field for the pattern formation can be locally and temporarily exceeded (see fig. S9B). This leads to the onset of the instability and the pattern formation locally, which disappear once the NP concentration drops again because of the concentration fluctuations. Last, the electroferrofluid patterns exist only with continuous energy feed and dissipation. This can be further directly quantified and linked to the pattern formation, as discussed in the following.

### Quantification of the dissipation and linking it to pattern formation

Direct and simple quantification of the dissipation is possible in the microelectrode cells, given in the steady state by *P* = *UI*_F_. Typically, for example, at *U* = 3 V and *I*_F_ ≈ 400 nA, the dissipated power is very modest *P* ~1.2 μW for a microelectrode cell with a surface area of ca. 1 cm^2^. The pattern formation upon application of the magnetic field (Figs. 3 and 4) leads to a measurable change in the Faradaic current Δ*I*_F_ (Fig. 5C) and thus a change in the dissipated power Δ*P* = *U*Δ*I*_F_ (Fig. 5D and fig. S11). The dissipated power increases upon pattern formation for both the labyrinthine-like and Rosensweig-like cases (Fig. 5D). This increase is caused by the redistribution of the charge-carrying NPs within the microelectrode cell and can be intuitively considered to be due to the formation of pathways with enhanced electrical conductivity between the positive and negative electrodes in the locations where the NP concentration increases. The nonlinearly increasing dissipation with the increasing *B*-field (Fig. 5D) can be understood to be linked to the nonlinear nature of the pattern formation (Figs. 3 and 4): Below the threshold *B*-field, there is no pattern formation (no change in the distribution of the charge-carrying NPs) and no change in the dissipation. In contrast, above the threshold *B*-field, the patterns appear, altering the distribution of the NPs and, hence, the electric current and dissipation change.

### Outlook toward detailed mechanistic understanding

In conclusion, we have demonstrated how to drive superparamagnetic NPs out of the thermodynamic equilibrium into nonequilibrium concentration gradient states using an electric field. The driving relies on proper charge control of the iron oxide NPs in the nonpolar solvent using AOT as the charge control agent. While charging of larger colloidal particles has been investigated earlier (*26*, *27*), the exact nature of the charge transfer pathways are complicated and remain to be identified for the small, ca. 10 nm diameter, iron oxide NPs used here.

Regarding the gradient formation, our experimental evidence suggests that two ingredients are critical. First, the NPs should carry charge of the same sign. Otherwise, both the theory (large Bjerrum length) and experimental evidence (Fig. 2B and fig. S4) suggest that the Coulombic aggregation of the oppositely charged NPs takes place. Second, when the charged NPs meet the electrode (with an opposite sign of electric charge), the interaction between the NP and the electrode has to be such that there is no irreversible binding of the NP on the electrode as this would lead to electrodeposition (as shown, e.g., by our control experiment in the aqueous environment; fig. S3). The small charge combined to the sterically protective oleic acid coating in the nonpolar environment are likely critical elements for preventing the electrodeposition.

It has been recently shown that silver NPs coated with dodecanethiol exhibit charging in nonpolar environment and can be driven with electric field into crystalline superlattices (*14*)—in contrast to the gradients observed in our experiments with iron oxide NPs. The experiments with silver NPs were carried out without large amounts of charge control agents, such as AOT here, suggesting the possibility that the numerous bulky AOT reverse micelles in our system contribute toward preventing formation of crystalline NP superlattices or other irregularly shaped solid structures. Full understanding of the gradient formation will likely require identification of the dominant electrochemical charge transfer processes and pathways and combining them to a continuum description of the statistical mechanics of the charge carriers and the electric field in the system.

### Outlook toward applications and other voltage-controlled nonequilibrium functionalities

The gradient states allow voltage-controlled magnetism. As classic ferrofluids are technically relevant (*19*), the present electroferrofluids suggest novel opportunities for functional materials and surfaces that can be controlled with both magnetic and electric fields. Voltage-controlled magnetism using charged magnetic NPs migrating in electric field also provide an interesting liquid-state analog to the ionic solid state devices using electric field–driven migration of ions in solid state materials to control the magnetic properties (*28*, *29*).

Other NPs with different functionalities than superparamagnetism can be envisioned to be similarly driven out of the homogeneous equilibrium state into concentration gradients to take advantage of their concentration-dependent properties and responses. This opens a generic platform for, e.g., voltage-controlled nonequilibrium plasmonics, photonics, and rheology based on the concentration-dependent optical response of plasmonic NPs (*30*), concentration-dependent photonic response of high-refractive index particles (*31*), and concentration-dependent jamming of load-bearing particles (*32*), paving way toward a new generation of functional colloidal materials and devices that can be tuned with an application of a small voltage. We also foresee that our approach can be generalized from a carrier liquid to an organogel matrix with suitable gel chemistry and porosity to allow migration of the NPs to create semisolid voltage-controlled gels for soft robotics, for example. Last, the extremely small power dissipation suggests feasible technological applications as, e.g., tunable optical devices or voltage-controlled inductors in mobile devices: A cm^2^-scale microelectrode cell drawing 400 nA current out of two standard 1.5 V AA batteries could, theoretically, reside in the dissipative steady-state continuously for over 500 years.

## MATERIALS AND METHODS

### Materials

Iron (III) chloride hexahydrate (FeCl_3_ **·** 6H_2_O, ≥99%; Sigma-Aldrich), iron (II) sulfate heptahydrate (FeSO_4_ **·** 7H_2_O, ≥99%, ACS reagent; Sigma-Aldrich), ammonium hydroxide (NH_4_OH, 28 to 30%, ACS reagent; Sigma-Aldrich), oleic acid (90% technical grade; Sigma-Aldrich), acetone (≥99.8%; Thermo Fisher Scientific), toluene (≥99.7%, ACS reagent; Sigma-Aldrich), *n*-dodecane (99%, anhydrous; Acros Organics), docusate sodium salt (AOT, ≥99%, anhydrous; Sigma-Aldrich), magnesium nitrate hexahydrate, [Mg(NO_3_)_2_ **·** 6H_2_O, 99%, ACS reagent; Sigma-Aldrich], and microelectrode cells with interdigitated ITO electrodes (Instec IPS10X10A040uNOPI) were used as received.

### Synthesis of stock dispersion of iron oxide NPs dispersed in toluene

Iron oxide NPs were synthesized by coprecipitation method (*33*) and stabilized with oleic acid in toluene. Briefly, 21.6 g of FeCl_3_ **·** 6H_2_O and 11.2 g of FeSO_4_ **·** 7H_2_O were mixed with 720 ml of ion-exchanged water (Milli-Q) in a plastic bottle (21040001, Thermo Fisher Scientific) under ambient conditions. Coprecipitation was initialized by adding 80 ml of NH_4_OH, followed by vigorous mechanical stirring for 5 min. The cap was opened occasionally to release the excess NH_3_. Thereafter, 25 g of oleic acid was added to the bottle, the cap was closed, and the container was continuously shaken in vigorous vertical movements for 10 min, with occasional opening of the cap in between. The container was left open in a fume hood overnight to complete the reactions and to release the NH_3_. The NPs were purified the next day. First, the NPs were sedimented by placing the container on top of a cube-shaped neodymium iron boron (NdFeB) magnet (5 cm by 5 cm by 5 cm) for 10 min. The supernatant was removed, and 400 ml of acetone was added to the mixture, followed by sonication at 37 kHz for 2 min to redisperse the sediment as aggregates. The NP aggregates were magnetically sedimented again within 2 to 3 min, resulting in a clear supernatant that was removed. Thereafter, 150 ml of toluene was added to redisperse the NPs and sonicated for 2 min. The NPs were precipitated by adding 200 ml of acetone, leading the color of dispersion to change from brown to dark grayish. The NPs were again magnetically sedimented within 2 min, and the supernatant was removed. Last, 50 ml of toluene was added, leading to spontaneous redispersion of the NPs by gently shaking the container. The NPs were then sonicated for 5 min with 37-kHz frequency to further assist the dispersion of NPs. The dispersion was left overnight in the fume hood to allow any remaining acetone to evaporate. The volume fraction of NPs in this stock dispersion was determined to be approximately 6.2% (text S1). The ferrofluid was also characterized with different techniques to determine the NPs size and composition (text S1).

### Preparation of electroferrofluids

A stock solution containing 300 mM of AOT in dodecane was prepared by mixing 4.01258 g of AOT and 19.7865 g of dodecane in a glass vial. The vial was sealed with parafilm to reduce moisture uptake from the ambient air. The vial was kept still for 2 days to ensure complete dissolution of AOT. Solutions with lower AOT concentration (e.g., 150 mM) were prepared from the 300 mM stock solution by diluting with pure dodecane. First, the stock dispersion of iron oxide NPs in toluene was sonicated (FB11207, Fisherbrand) for 10 min at 37 kHz and filtered through a polytetrafluoroethylene membrane with 0.45-mm pores (Titan3, Thermo Fisher Scientific). One milliliter of the sonicated and filtered dispersion was mixed with 1 ml of a solution of AOT in dodecane with desired AOT concentration (e.g., 150 mM) in a glass vial. The vial was left open in a fume hood for ca. 36 hours until toluene was evaporated, which was confirmed with Fourier transform infrared analysis (Nicolet 380, Thermo Fisher Scientific) by following the disappearance of the C-C vibration mode of the aromatic ring of toluene (reference peak at 1496 cm^−1^; fig. S1E). Last, the moisture content was adjusted by placing the vial for ca. 15 to 30 hours in a desiccator cabinet (467-0130, VWR) at room temperature with 55% relative humidity (created with a saturated aqueous solution of magnesium nitrate hexahydrate), resulting in the final electroferrofluid. The final electroferrofluid composition, i.e., volume fraction of NPs and AOT molarity, were estimated to be only slightly lower than in the stock NP dispersion and stock AOT solution (text S2). Throughout the text, we refer to nominal AOT concentrations used in preparation of the electroferrofluid, which are close to the true values (text S2). The electroferrofluid magnetic properties were also characterized (text S2).

### Filling microelectrode cells and making electrical connections

Electrical connections to the microelectrode cells were done using thin insulated copper wires by attaching them to the cells with silver paste (OK-SPI, SPI Supplies). The paste was dried at room temperature for at least 2 hours. The microelectrode cells were filled with the electroferrofluid using capillary action. The electroferrofluid was sonicated and filtered (similarly as during electroferrofluid preparation) just before filling the microelectrode cell for experiments on microscopic pattern formation (Figs. 2D, 3, 4, and 5 and figs. S5, S7, S8, and S9). In other experiments (Figs. 1E and 2B and fig. S4), the electroferrofluid was used without further sonication or filtering, and small amounts of NP aggregates can thus be seen in the microscopy images.

### Construction of the experimental setup for microscopic observation of the electroferrofluid under controlled electric and magnetic fields

#### Electric field

A high-resistance meter/electrometer (B2987A, Keysight) was used to apply electric field across the sample and simultaneously measure the current, typically at 0.1 s intervals. Before each measurement, the electroferrofluid sample was ensured to be in the thermodynamic ground state by applying 0 V for 40 s, during which the electric current decayed close to 0 A, and a homogeneous NP concentration was established. For experiments with constant electric field (Figs. 1D; 2 B, D to F; 3, B, C, and E; 4, B, C, and E; and 5A and figs. S4, S5, S6, S8, S10, and S12), a constant voltage step was applied to the sample for 150 s. Electric potential and electric field inside the microelectrode cells (fig. S2, D and E) were simulated using finite element method (COMSOL Multiphysics 5.4). Electric Currents interface was used together with manufacturer-specified cell geometry (fig. S2C) that were confirmed (fig. S2B) using optical microscopy (50×/0.55, Zeiss Z1 Imager and Zeiss Epiplan-Neofluar). The following conductivity and relative permittivity values were used in the simulation, respectively: glass: 1 × 10^−20^ S/m, 7.3; sample space (dodecane): 1 × 10^−8^ S/m, 2.0; ITO electrodes: 5 × 10^−6^ S/m, 3.6.

#### Magnetic field

A pair of small electromagnet coils (11801523 and 11801524, GMW) connected to a DC power supply (9205, BK Precision) was used to generate uniform magnetic fields (fig. S7, A and B). The magnetic field between the coils was measured using a three-axis Teslameter (3MTS, Senis; fig. S7, C to E). For experiments with constant magnetic fields (Figs. 3 B, C, and E; 4 B, C, and E; and 5A and figs. S8, S10, S11, and S12), the magnetic field was applied 30 s after the electric field for a duration of ca. 90 s. Represented images (Figs. 3C, 4C, and 5A and figs. S8, A to C, S10, A to C, and S12) demonstrate an image acquired 60 s after applying the magnetic field. For experiments with different NP volume fractions (fig. S9), the electric field was stabilized for 20 s, after which the magnetic field was applied as a staircase from 0 mT to *B*_c_ with 1-mT intervals, and each *B*-field was kept for 20 s. The representative images (fig. S9) were taken 15 s after each update in the *B*-field.

#### Microscopy

The microelectode cell was illuminated in transmitted light configuration using an light-emitting diode light source (MCWHLP1, Thorlabs), a collimator (COP4-A, Thorlabs), and a diffuser. Imaging was done with a 10× finite-conjugate objective lens (Nikon 10×/0.25 160/−WD5.6) or a 4× finite-conjugate objective lens (Nikon 4×/0.25 160/−WD25) connected to a five-megapixel gray scale camera (MC050MG-SY, Ximea). Image length scale was calibrated using a calibration target (R1L3S2P, Thorlabs). Images were acquired using software provided by the camera manufacturer (xiCamTool 4.28, Ximea). In most experiments, images were acquired at 100 frames per second (fps) with averaging of five consecutive frames to reduce noise, resulting in final acquisition rate of 20 fps. The Rosensweig-like instabilities (Fig. 4, B and C) were imaged at 5 fps with averaging five consecutive images, leading to 1 fps, and the control experiments in polar solvent (fig. S3) at 30 fps with no image averaging. Images used for the voltage-controlled magnetism analysis (Fig. 2G) were acquired after 20 s stabilization at each voltage without averaging.

### *I*-*V* measurements

*I*-*V* measurements (Fig. 2A) were carried out using the same setup used for the main experiments. A voltage staircase of 0 → +3 → −3 → 0V at 0.1 V intervals was applied, and electric current was measured at each voltage after 30 s stabilization time. The measurement was performed three times in different microelectrode cells for 150 mM AOT in dodecane and the electroferrofluid and once for the iron oxide NPs in dodecane (0 mM AOT). Similarly, the images used for the analysis of the voltage-controlled magnetism (Fig. 2G) were collected by applying a voltage staircase from 0 to 4 V, with 0.1 V intervals, and the sample was allowed to stabilize for 20 s before taking each image.

### Image acquisition, processing, and analysis

#### Image acquisition, bit-depth conversion, and contrast adjustment

Microscopy images were acquired with 12-bit analog-to-digital conversion and saved as 12/16-bit gray scale tagged image file format (TIFF) files. These high-bit images (raw data) were converted to 8-bit gray scale images with simultaneous histogram adjustment with ImageJ2/Fiji (*34*, *35*) to improve contrast of the NP gradients and patterns (see fig. S12 for typical examples of raw and processed images). All shown microscopy images have been adjusted unless otherwise stated in the text.

#### Analysis of voltage-controlled magnetism

The raw 12/16-bit images of the steady state (*U* > 0 V; fig. S5A and Fig. 2D) were divided pixel-by-pixel with the image of the thermodynamic ground state (*U* = 0 V) with ImageJ/Fiji. This results in images consisting of real numbers close to 1.0 for each pixel (fig. S5B), which were saved as TIFF files with real (32-bit) values. These images were opened in MATLAB to calculate the natural logarithm (fig. S5C and Fig. 2E). From these images, the profiles (Fig. 2F) were obtained by averaging along the direction of the electrodes.

#### Kymographic analysis

Kymographs (Figs. 3E and 4E) were extracted using ImageJ2/Fiji by opening the image time series as an image stack, defining a one-pixel-wide region of interest (a line) along the electrodes and between a pair of a positive and a negative electrode, at a distance of *d*/4 away from the edge of the positive electrode. Kymographs were then generated using the reslice command of ImageJ2/Fiji.

#### Fourier analysis

Fourier analysis of the transition from a steady-state gradient to a steady-state pattern (figs. S8E and S10E) was carried out using a custom MATLAB code calculating the fast Fourier transform (FFT) of each row of pixels along the electrodes separately, followed by averaging the absolute values of the FFT over all pixel lanes of the image. The periodicity of patterns (Figs. 3D and 4D) was obtained from the location of the peak in the Fourier transformation by applying a Gaussian fit.

### Analysis of dissipation

The change in power dissipation upon pattern formation (Fig. 5D) was obtained as follows. First, the change in electric current upon application of magnetic field (resulting in pattern formation) was extracted from *I*(*t*) by performing a baseline fitting and subtraction (fig. S11, A to D). The steady-state change in the current (Δ*I*) was then obtained after the change in current had stabilized (fig. S11E). Multiplication with the applied voltage led to the change in dissipation (Fig. 5D). This analysis also yields other useful information, such as the characteristic time scales of the growth and relaxation of the increase in dissipation (fig. S11F) (*36*–*39*).

## References

[1] M. C. Cross, P. C. Hohenberg, Pattern formation outside of equilibrium. Rev. Mod. Phys. 65, 851–1112 (1993).

[2] B. A. Grzybowski, K. Fitzner, J. Paczesny, S. Granick, From dynamic self-assembly to networked chemical systems. Chem. Soc. Rev. 46, 5647–5678 (2017). 28703815 10.1039/c7cs00089h

[3] A. Sorrenti, J. Leira-Iglesias, A. J. Markvoort, T. F. A. De Greef, T. M. Hermans, Non-equilibrium supramolecular polymerization. Chem. Soc. Rev. 46, 5476–5490 (2017). 28349143 10.1039/c7cs00121e PMC5708531

[4] M. Grzelczak, L. M. Liz-Marzán, R. Klajn, Stimuli-responsive self-assembly of nanoparticles. Chem. Soc. Rev. 48, 1342–1361 (2019). 30688963 10.1039/c8cs00787j

[5] G. M. Whitesides, B. Grzybowski, Self-assembly at all scales. Science 295, 2418–2421 (2002). 11923529 10.1126/science.1070821

[6] B. A. Grzybowski, H. A. Stone, G. M. Whitesides, Dynamic self-assembly of magnetized, millimetre-sized objects rotating at a liquid-air interface. Nature 405, 1033–1036 (2000). 10890439 10.1038/35016528

[7] J. V. I. Timonen, M. Latikka, L. Leibler, R. H. A. Ras, O. Ikkala, Switchable static and dynamic self-assembly of magnetic droplets on superhydrophobic surfaces. Science 341, 253–257 (2013). 23869012 10.1126/science.1233775

[8] J. Boekhoven, W. E. Hendriksen, G. J. M. Koper, R. Eelkema, J. H. van Esch, Transient assembly of active materials fueled by a chemical reaction. Science 349, 1075–1079 (2015). 26339025 10.1126/science.aac6103

[9] S. Maiti, I. Fortunati, C. Ferrante, P. Scrimin, L. J. Prins, Dissipative self-assembly of vesicular nanoreactors. Nat. Chem. 8, 725–731 (2016). 27325101 10.1038/nchem.2511

[10] R. Klajn, P. J. Wesson, K. J. M. Bishop, B. A. Grzybowski, Writing self-erasing images using metastable nanoparticle “inks”. Angew. Chem. Int. Ed. Engl. 48, 7035–7039 (2009). 19533698 10.1002/anie.200901119

[11] I. Lagzi, B. Kowalczyk, D. Wang, B. A. Grzybowski, Nanoparticle oscillations and fronts. Angew. Chem. Int. Ed. Engl. 49, 8616–8619 (2010). 20886492 10.1002/anie.201004231

[12] M. Sawczyk, R. Klajn, Out-of-Equilibrium aggregates and coatings during seeded growth of metallic nanoparticles. J. Am. Chem. Soc. 139, 17973–17978 (2017). 29193964 10.1021/jacs.7b09111

[13] R. K. Grötsch, A. Angı, Y. G. Mideksa, C. Wanzke, M. Tena-Solsona, M. J. Feige, B. Rieger, J. Boekhoven, Dissipative self-assembly of photoluminescent silicon nanocrystals. Angew. Chem. Int. Ed. Engl. 57, 14608–14612 (2018). 30040877 10.1002/anie.201807937

[14] Y. Yu, D. Yu, C. A. Orme, Reversible, tunable, electric-field driven assembly of silver nanocrystal superlattices. Nano Lett. 17, 3862–3869 (2017). 28511013 10.1021/acs.nanolett.7b01323

[15] R. Kemp, R. Sanchez, K. J. Mutch, P. Bartlett, Nanoparticle charge control in nonpolar liquids: Insights from small-angle neutron scattering and microelectrophoresis. Langmuir 26, 6967–6976 (2010). 20092312 10.1021/la904207x

[16] M. Karvar, F. Strubbe, F. Beunis, R. Kemp, N. Smith, M. Goulding, K. Neyts, Charging dynamics of aerosol OT inverse micelles. Langmuir 31, 10939–10945 (2015). 26375733 10.1021/acs.langmuir.5b01677

[17] A. I. Bulavchenko, D. N. Pletnev, Electrophoretic concentration of nanoparticles of gold in reversed micellar solutions of AOT. J. Phys. Chem. C 112, 16365–16369 (2008).

[18] G. E. Pradillo, H. Karani, P. M. Vlahovska, Quincke rotor dynamics in confinement: Rolling and hovering. Soft Matter 15, 6564–6570 (2019). 31360980 10.1039/c9sm01163c

[19] X. Zhang, L. Sun, Y. Yu, Y. Zhao, Flexible ferrofluids: Design and applications. Adv. Mater. 31, 1903497 (2019). 10.1002/adma.201903497 31583782

[20] R. E. Rosensweig, *Ferrohydrodynamics* (Cambridge Univ. Press, 1985).

[21] M. D. Cowley, R. E. Rosensweig, The interfacial stability of a ferromagnetic fluid. J. Fluid Mech. 30, 671–688 (1967).

[22] C. Gollwitzer, G. Matthies, R. Richter, I. Rehberg, L. Tobiska, The surface topography of a magnetic fluid: A quantitative comparison between experiment and numerical simulation. J. Fluid Mech. 571, 455–474 (2007).

[23] R. E. Rosensweig, M. Zahn, R. Shumovich, Labyrinthine instability in magnetic and dielectric fluids. J. Magn. Magn. Mater. 39, 127–132 (1983).

[24] A. J. Dickstein, S. Erramilli, R. E. Goldstein, D. P. Jackson, S. A. Langer, Labyrinthine pattern formation in magnetic fluids. Science 261, 1012–1015 (1993). 17739618 10.1126/science.261.5124.1012

[25] M. Igonin, A. Cebers, Labyrinthine instability of miscible magnetic fluids. Phys. Fluids 15, 1734–1744 (2003).

[26] G. N. Smith, J. E. Hallett, J. Eastoe, Celebrating Soft Matter’s 10th Anniversary: Influencing the charge of poly(methyl methacrylate) latexes in nonpolar solvents. Soft Matter 11, 8029–8041 (2015). 26369696 10.1039/c5sm01190f

[27] S. K. Sainis, V. Germain, C. O. Mejean, E. R. Dufresne, Electrostatic interactions of colloidal particles in nonpolar solvents: Role of surface chemistry and charge control agents. Langmuir 24, 1160–1164 (2008). 18062711 10.1021/la702432u

[28] U. Bauer, L. Yao, A. J. Tan, P. Agrawal, S. Emori, H. L. Tuller, S. Van Dijken, G. S. D. Beach, Magneto-ionic control of interfacial magnetism. Nat. Mater. 14, 174–181 (2015). 25401920 10.1038/nmat4134

[29] A. Molinari, H. Hahn, R. Kruk, Voltage-control of magnetism in all-solid-state and solid/liquid magnetoelectric composites. Adv. Mater. 31, 1806662 (2019). 10.1002/adma.201806662 30785649

[30] V. Amendola, R. Pilot, M. Frasconi, O. M. Maragò, M. A. Iatì, Surface plasmon resonance in gold nanoparticles: A review. J. Phys. Condens. Matter 29, 203002 (2017). 28426435 10.1088/1361-648X/aa60f3

[31] Z. Cai, Z. Li, S. Ravaine, M. He, Y. Song, Y. Yin, H. Zheng, J. Teng, A. Zhang, From colloidal particles to photonic crystals: Advances in self-assembly and their emerging applications. Chem. Soc. Rev. 50, 5898–5951 (2021). 34027954 10.1039/d0cs00706d

[32] V. Trappe, V. Prasad, L. Cipelletti, P. N. Segre, D. A. Weitz, Jamming phase diagram for attractive particles. Nature 411, 772–775 (2001). 11459050 10.1038/35081021

[33] R. Massart, Preparation of aqueous magnetic liquids in alkaline and acidic media. IEEE Trans. Magn. 17, 1247–1248 (1981).

[34] J. Schindelin, I. Arganda-Carreras, E. Frise, V. Kaynig, M. Longair, T. Pietzsch, S. Preibisch, C. Rueden, S. Saalfeld, B. Schmid, J. Y. Tinevez, D. J. White, V. Hartenstein, K. Eliceiri, P. Tomancak, A. Cardona, Fiji: An open-source platform for biological-image analysis. Nat. Methods 9, 676–682 (2012). 22743772 10.1038/nmeth.2019 PMC3855844

[35] C. T. Rueden, J. Schindelin, M. C. Hiner, B. E. DeZonia, A. E. Walter, E. T. Arena, K. W. Eliceiri, ImageJ2: ImageJ for the next generation of scientific image data. BMC Bioinformatics 18, 529 (2017). 29187165 10.1186/s12859-017-1934-z PMC5708080

[36] H. Okudera, K. Kihara, T. Matsumoto, Temperature dependence of structure parameters in natural magnetite: Single crystal x-ray studies from 126 to 773 K. Acta Crystallogr. Sect. B Struct. Sci. 52, 450–457 (1996).

[37] J.-E. Jørgensen, L. Mosegaard, L. E. Thomsen, T. R. Jensen, J. C. Hanson, Formation of γ-Fe_2_O_3_ nanoparticles and vacancy ordering: An in situ X-ray powder diffraction study. J. Solid State Chem. 180, 180–185 (2007).

[38] D. L. A. de Faria, S. Venâncio Silva, M. T. de Oliveira, Raman microspectroscopy of some iron oxides and oxyhydroxides. J. Raman Spectrosc. 28, 873–878 (1997).

[39] J. Coey, *Magnetism and Magnetic Materials* (Cambridge Univ. Press, 2010).

