# Supplementary material for: Electroferrofluids with nonequilibrium voltage-controlled magnetism, diffuse interfaces, and patterns
Source: Sci Adv. 2021 Dec 22;7(52):eabi8990. doi: 10.1126/sciadv.abi8990 (PMC8694619; doi:10.1126/sciadv.abi8990)
Supplement: Supplementary file 1 — Supplementary Text S1 to S4 Figs. S1 to S12 Tables S1 to S4 Legends for movies S1 to S6 [file sciadv.abi8990_sm.pdf]

Science Advances
AAAS

Supplementary Materials for

Electroferrofluids with nonequilibrium voltage-controlled magnetism, diffuse interfaces, and patterns

Tomy Cherian, Fereshteh Sohrabi, Carlo Rigoni, Olli Ikkala, Jaakko V. I. Timonen*

*Corresponding author. Email: jaakko.timonen@aalto.fi

Published 22 December 2021, *Sci. Adv.* **7**, eabi8990 (2021)
DOI: 10.1126/sciadv.abi8990

The PDF file includes:

Supplementary Text S1 to S4
Figs. S1 to S12
Tables S1 to S4
Legends for movies S1 to S6

Other Supplementary Material for this manuscript includes the following:

Movies S1 to S6

Supplementary text

Supplementary text S1. Characterization of the iron oxide NPs

Determination of the volumetric composition of the stock dispersion of iron oxide NPs in toluene: Volume fraction of NPs (solid content) in a ferrofluid (ϕ_{F}) was estimated by measuring and analyzing the mass of solid NPs (m_{S}) in a known mass of the ferrofluid (m_{F}) after evaporating the carrier solvent (toluene, with mass m_{T}). Briefly, approximately V_{F} = 100 μl of the ferrofluid was transferred into a 1.5 ml glass vial with known weight using a pipette (Eppendorf Z683809) and the mass of the vial with ferrofluid was measured, yielding the mass of the ferrofluid m_{F}. The vial with ferrofluid was left open in a fume hood overnight to allow most of the toluene to evaporate. Remaining toluene (and other volatiles that we assumed they have negligible amounts) were removed in oven (Memmert UF30) at 70 $^{\circ}$C for 30 hours. The remaining mass determined with analytical balance was m_{S}, thus yielding mass of evaporated toluene as $m_{\mathrm{T}} = m_{\mathrm{F}} - m_{\mathrm{S}}$. The volume fraction of solid content in the toluene ferrofluid is obtained as:

$$\phi_{\mathrm{F}} = \frac{V_{\mathrm{S}}}{V_{\mathrm{F}}} = \frac{V_{\mathrm{F}} - V_{\mathrm{T}}}{V_{\mathrm{F}}} = 1 - \frac{V_{\mathrm{T}}}{V_{\mathrm{F}}} = 1 - \frac{m_{\mathrm{T}}}{\rho_{\mathrm{T}}}\frac{\rho_{\mathrm{F}}}{m_{\mathrm{F}}}, \tag{S1}$$

where ρ_{F} is the density of the ferrofluid and ρ_{T} is the density of toluene. We obtained approximate values for these by measuring the mass of a known volume of the liquids using an analytical balance and a positive displacement pipette (Eppendorf multipette E3x): $\rho_{\mathrm{F}} = 1.108 \pm 0.007$ g/ml and $\rho_{\mathrm{T}} = 0.854 \pm 0.001$ g/ml. The volume fraction measurement was performed for three samples (table S1). The average volume fraction of NPs calculated from the three measurements is $\phi_{\mathrm{F}} \approx$ 6.2 % and the volume fraction of toluene is thus $\phi_{\mathrm{T}} \approx$ 93.8 %. We note that in this approach the free surfactants (non-volatile) are also included in the volume fraction of NPs. We also note the relative error of ca. 1.3 % when comparing measured toluene density and literature value (0.865 g/ml) and used the measured value that we assume to have similar systematic error as the ferrofluid density.

Table S1: Data used to calculate the volume fraction of the solid content in the ferrofluid in toluene

Sample number	m_{F} (mg)	m_{S} (mg)	m_{T} (mg)	ϕ_{F} (%)
1	101.2	28.0	73.2	6.1
2	100.9	28.0	72.9	6.3
3	100.6	27.8	72.8	6.1

Transmission electron microscopy (TEM): NP morphology and size distribution were determined using a transmission electron microscope (JEOL JEM-2800, 200 kV). TEM sample was prepared by diluting the NP stock dispersion with toluene approximately in ratio 1:1000 and pipetting a droplet of the diluted dispersion on a TEM grid with a holey carbon film (Agar Scientific S147-4) and allowing the toluene to evaporate. From individual TEM images (fig. S1A), the NP size distribution (fig. S1B) was determined using image analysis software ImageJ2/Fiji by measuring

the diameter of each particle manually along a randomly chosen axis. Approximately 350 particles were measured, and the resulting histogram was interpolated with a log-normal distribution, given by:

$$y = y_0 + \frac{A}{xS\sqrt{2\pi}} \exp\left(-\frac{(\ln(x) - M)^2}{2S^2}\right). \tag{S2}$$

The best fit was obtained with parameters given in Table S1, from which the mean (μ) and standard deviation (σ) were obtained as:

$$\mu = e^{M+S^2/2}, \ \ \sigma^2 = e^{S^2+2M}\left(e^{S^2} - 1\right). \tag{S3}$$

resulting in μ = 8.31 ± 0.06 nm and σ = 2.64 ± 0.04 nm.

Table S2: Parameter of the log-normal fit of the NP distribution measured from TEM images.

y_0	A	M	S
0 ± 1	751 ± 23	2.069 ± 0.007	0.310 ± 0.007

X-ray diffraction (XRD): Crystallographic structure of the iron oxide NPs was studied using X-ray diffraction (Rigaku SmartLab). Dry NP powder was prepared by allowing 500-1000 μl of stock NP dispersion to dry in a ceramic evaporating dish overnight. The dry powder was collected and deposited on a standard microscope glass slide. Large NP aggregates were crushed to a fine powder using another microscope slide. This fine powder on a glass slide was then used to measure XRD. The X-ray data was collected in the 2θ range of 5 - 90° at a scanning speed of 1 degree/min with scan step of 0.04^{o} (fig. S1C) and then compared with magnetite and maghemite XRD reference data (*36, 37*). Scherrer analysis was carried out using the six most intense diffraction peaks, yielding an average crystallite size of 7.1 ± 1.5 nm.

Raman spectroscopy: Crystallographic structure of the iron oxide NPs were studied using Raman spectroscopy (Horiba LabRAM HR). Similar sample as for XRD was used. Measurements were carried out with 632.8 nm laser (HeNe laser) in the range of 50 - 1800 cm^{-1}. The laser beam was focused on the NP powder using a 50x objective lens. Data was collected for 120 seconds and averaged over two cycles. The laser power was limited to below 0.6 mW to avoid beam damage (fig. S1D). Data were compared with reference locations of the characteristic magnetite peak (ca. 660 cm^{-1}) and maghemite peaks (300 - 500 cm^{-1}) (*38*).

Supplementary text S2. Characterization of the electroferrofluid (150 mM AOT)

Determination of the volumetric composition of electroferrofluid (150 mM AOT): Standard electroferrofluid with 150 mM AOT was prepared by mixing V_F = 100 μl of ferrofluid (Supplementary Materials S1) and $V_{AOT/DD}$ = 100 μl of 150 mM AOT in dodecane, and allowing toluene to evaporate completely at room temperature, leading to final electroferrofluid with volume V_{EF}. Assuming no loss of dodecane or significant moisture uptake during the evaporation,

the volume fraction of NPs (ϕ_{NP}) and the volume fraction of AOT (ϕ_{AOT}) in the final electroferrofluid can be calculated as:

$$\phi_{NPs} = \frac{V_{NPs}}{V_{EF}} = \frac{V_F \phi_F}{V_{AOT/DD} + V_F \phi_F}, \quad (S4)$$

and

$$\phi_{AOT} = \frac{V_{AOT}}{V_{EF}} = \frac{V_{AOT/DD} \phi_{AOT/DD}}{V_{AOT/DD} + V_F \phi_F}. \quad (S5)$$

Inserting the above values to the equations together with ϕ_F = 6.2 % (see Supplementary text section S1) and the volume fraction of AOT in 150 mM AOT in dodecane, that was calculated as: $\phi_{AOT/DD} = \frac{0.15 \text{ mol/l} \ . \ 444.5583 \text{ g/mol}}{1100 \text{ g/l}}$ ≈ 6.1 %, we obtain ϕ_{NPs} ≈ 5.8 % and ϕ_{AOT} ≈ 5.7 %. Due to the several approximations and experimental uncertainties, we conclude that the final electroferrofluid with 150 mM AOT contains (volumetrically) approximately 6 % of iron oxide NPs (including oleic acid stabilizer), 6 % of charge control agent AOT, and 88 % of dodecane. In addition, there is a small amount of water in the electroferrofluid that originates from preparation of the electroferrofluid under ambient conditions and incubation in the humidity chamber. The volume fraction of water was estimated to be approximately 0.5 - 1.0 % using Karl Fischer titration (Mettler Toledo Coulometric KF Titrator C30S).

Magnetic properties of the electroferrofluid: Magnetic properties of the electroferrofluid (150 mM AOT) were measured with a vibrating sample magnetometer (QuantumDesign PPMS VSM). Sample was prepared by filling a 3 cm long capillary tube (ca. 0.23 mm inner radius) with ca. 0.5 µl of the electroferrofluid. The capillary was sealed with UV curable adhesive (Norland Optical Adhesive 61) and cured under UV lamp (Thorlabs Solis 365C).
The data collected with the magnetometer as described in the Methods were then analyzed as follows. The paramagnetic background from sample holder was subtracted from the measured data performing a linear fit of the data at the highest field. Positive half of the magnetic loop of the electroferrofluid is shown in fig. S1F. The data were interpolated with the Langevin function as:

$$M = nm \left[\coth\left(\frac{mB}{k_B T}\right) - \left(\frac{mB}{k_B T}\right)^{-1} \right], \quad (S6)$$

where M is the magnetization of the sample, B is the applied magnetic field, k_B is the Boltzmann constant, T is the temperature, n is the concentration of the particles (measured in m^{-3}), and m is the average NP moment (measured in Am^2). The results of the interpolation are summarized in table S3.

Table S3: NP magnetic moment (m) and concentration (n) in the electroferrofluid (EF).

m_{EF} (Am^2)	n_{EF} (m^{-3})
$(2.03 \pm 0.08) \cdot 10^{-19}$	$(4.4 \pm 0.2) \cdot 10^{22}$

Comparing the magnetic moment of the NPs with the magnetic moment of the bulk magnetic material (M_b) can allow us to derive different useful quantities. After simple calculations from the Langevin equation we can write that:

$$d = \left(\frac{6m}{\pi M_b}\right)^{\frac{1}{3}}, \quad \phi = Vn = \frac{\pi}{6} d^3 n, \quad M_s = \phi M_b, \quad \chi = \frac{\mu_0 m M_s}{3 k_B T}, \qquad \text{(S7)}$$

where d is the magnetic core diameter, ϕ is the volume fraction of the magnetic material (iron oxide) in the electroferrofluid, $M_b \approx 0.48$ MAm^{-1} is saturation magnetization of magnetite (*39*), M_s is the saturation magnetization of the fluid and χ is its magnetic susceptibility. The calculated values for the electroferrofluid are shown in table S4. We note that the errors indicated for d_{EF}, m_{EF} and n_{EF} (tables S3 and S4) indicate uncertainties of the Langevin fit, rather than the widths of the corresponding distributions. Langevin model also assumes monodisperse non-interacting NPs, which is not the case here, so that the fitted parameter values should be considered only as effective parameters for the electroferrofluid and as approximations for the nanoparticles themselves.

Table S4. Results of the calculation with formulas S4 for the electroferrofluid (EF).

d_{EF} (nm)	ϕ_{EF} (%)	$M_{S/EF}$ (kA/m)	χ_{EF}
9.3 ± 0.4	1.9 ± 0.1	8.93 ± 0.05	0.18 ± 0.01

One can immediately notice that the volume fraction of the magnetic material ϕ_{EF} is lower than the volume fraction of the solid content ϕ_{EF} calculated previously. This is due to the fact that volume fraction ϕ_{EF} calculated here corresponds only to the magnetic core of NPs in electroferrofluid; whereas, the non-magnetic oleic acid shell that encapsulates the magnetic core also contributes to solid content of NPs in calculations done in the previous section.

Supplementary text S3. Control experiment with negatively charged superparamagnetic NPs in polar solvent (water)

An aqueous ferrofluid consisting of maghemite NPs stabilized with citric acid (electrostatic double layer forces) was synthesized as described earlier (*33*). A microelectrode cell was activated in a plasma oven (Henniker Plasma HPT-100) for 5 minutes to increase the hydrophilicity and to enable filling with the aqueous ferrofluid. The sample was imaged, and electric and magnetic fields were applied with the same setup as in experiments with the electroferrofluid samples (fig. S3).

Supplementary text S4. Light absorption concentration analysis with the Beer-Lambert law.

The electrically controlled dissipative build-up of concentration gradients of superparamagnetic NPs allows electrically controlled non-equilibrium magnetic responses. In the first approximation, the magnetic susceptibility χ and the saturation magnetization M_S of a NP dispersion is linearly proportional to the NP concentration c. It is difficult to perform direct local magnetic measurements on the voltage-controlled state, and we thus rely on approximate quantification by analyzing optical transmittance changes through the sample that reflect on the NP concentration. Approximately, by the Beer-Lambert law, light intensity transmitted through the microelectrode cell in any location depends on the local concentration of NPs as $I \propto e^{-\alpha hc}$ where α is extinction coefficient of the NPs. It follows that we can define for each spatial location (pixel) a normalized change in NP concentration (ΔC) that is proportional to the difference of the non-equilibrium NP concentration (c) and the equilibrium concentration (c_0), and at the same time can be calculated from the ratio between the transmitted light intensity in the non-equilibrium state (I) and in the equilibrium state (I_0) as

$$\Delta C \approx \alpha h(c - c_0) \approx -\ln(I/I_0), \quad \text{(S8)}$$

considering equilibrium magnetic susceptibility and saturation magnetization of NPs as χ_0 and M_{S0}, for non-interacting NPs, $\chi \propto c$ and $M_S \propto c$, and thus the changes in the magnetic susceptibility and saturation magnetization are $\Delta\chi = \chi - \chi_0 \propto \Delta C$ and $\Delta M_S = M_S - M_{S0} \propto \Delta C$. Therefore, ΔC is a fingerprint parameter linearly proportional to the local change in both the susceptibility and the saturation magnetization.

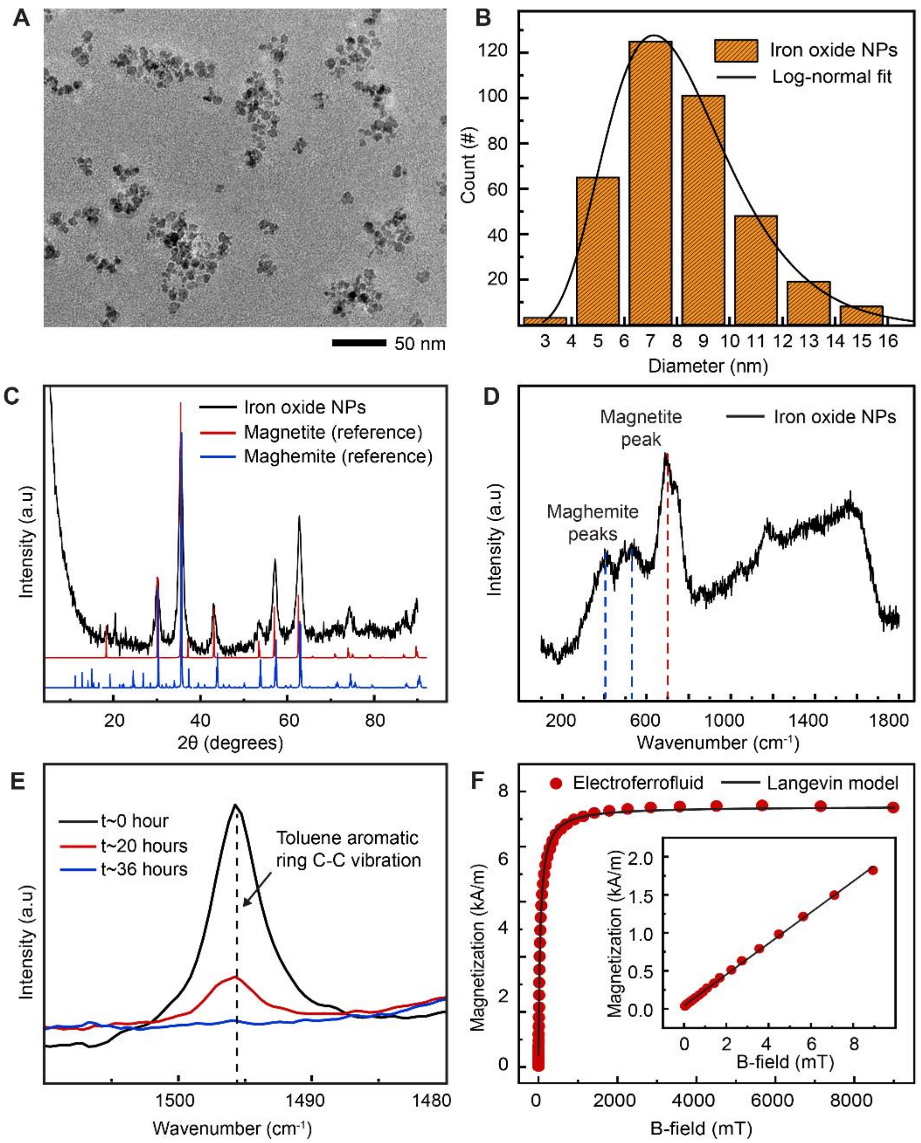

Fig. S1.

Characterization of superparamagnetic NPs and the electroferrofluid. **(A)** A typical TEM image of the iron oxide NPs stabilized with oleic acid. **(B)** NP diameter histogram obtained from TEM images with log-normal fit. **(C)** Powder XRD diffractogram of iron oxide NPs and references. **(D)** Raman spectrum of iron oxide NPs and references for pure magnetite and maghemite peaks. **(E)** FTIR spectrum of mixture of iron oxide NPs in toluene and 150 mM AOT in dodecane for three data points during the evaporation of toluene at room temperature. **(F)** Magnetization curve of the electroferrofluid (150 mM AOT) and the best fit of Langevin model. The inset is a zoom of the data near the origin.

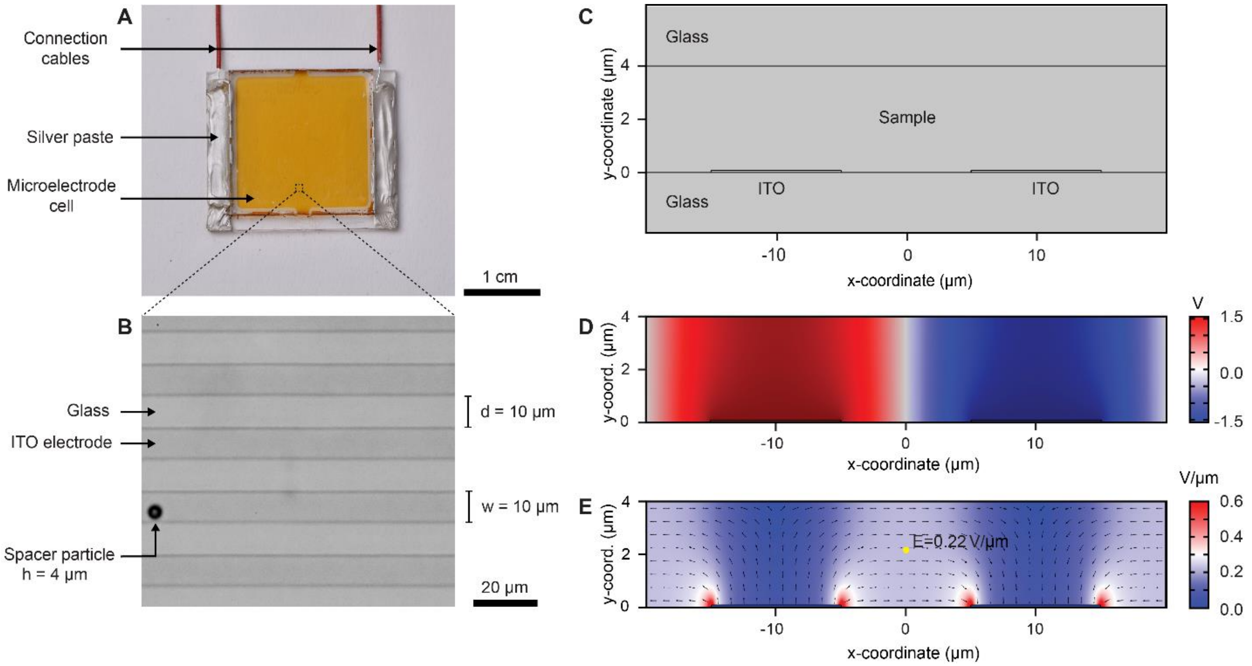

Fig. S2.

Microelectrode cell geometry and the electric field within. **(A)** Photograph of a microelectrode cell filled with electroferrofluid. **(B)** Micrograph of a microelectrode cell, showing lanes of interdigitated electrodes. **(C)** Cross-sectional drawing of the microelectrode cell based on manufacturer specifications. **(D)** Electric potential and **(E)** electric field inside the microelectrode cell when a potential difference of 3 V is applied between neighboring ITO electrodes with edge-to-edge distance d = 10 µm (COMSOL simulation). The electric field is approximately uniform between the electrodes (0.22 V/µm), with the exception of areas near the electrode corners where it is significantly stronger. In the main text, all quoted electric field strengths are nominal values calculated as $E = U/d$ (0.3 V/µm in this COMSOL example), corresponding to the average field strength between two neighboring electrodes on the bottom the microelectrode cell.

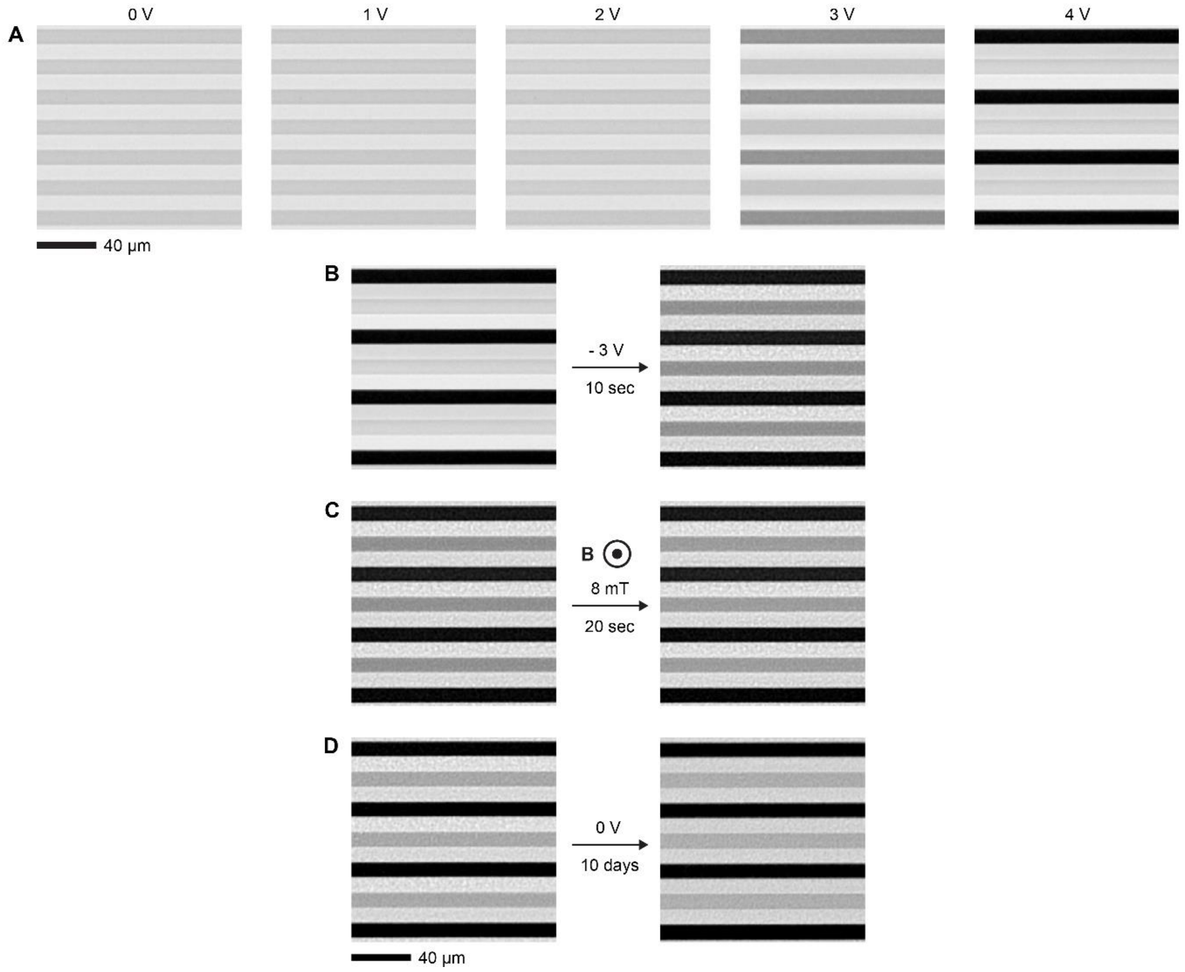

Fig. S3.

Control experiment with negatively charged superparamagnetic NPs in polar solvent (water). (A) Microscopy images of a microelectrode cell filled with aqueous dispersion of negatively charged iron oxide NPs at different applied voltages. **(B-D)** Microscopy images of the same microelectrode cell, showing no significant response of the sample to *B*, the change in polarity of the electric field, **(C)** application of magnetic field, or **(D)** long-term relaxation in absence of external electric and magnetic field.

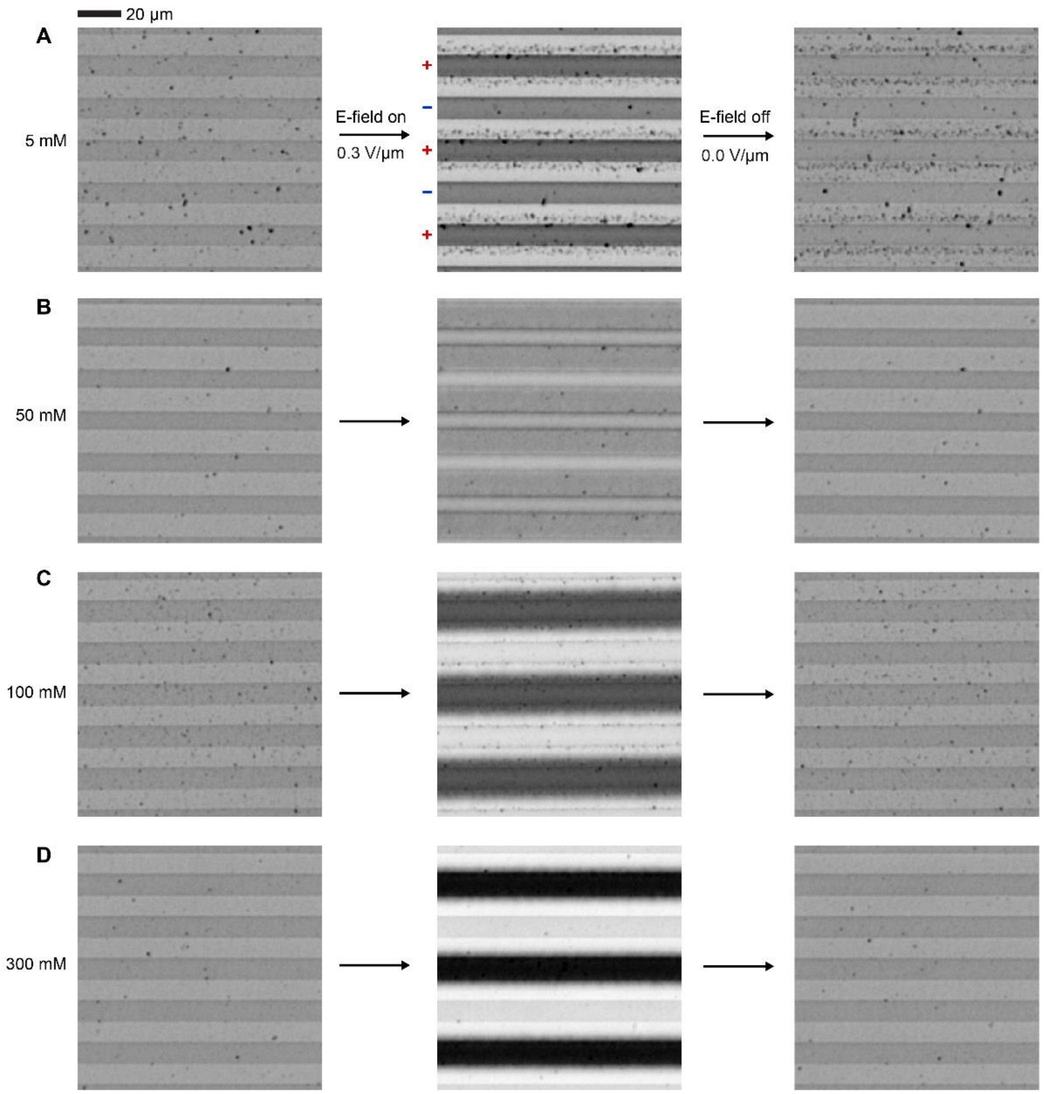

Fig. S4.

Effect of charge control agent (AOT) concentration on electroferrofluid behavior. Each row **(A-D)** contains three microscopy images of electroferrofluid of varying AOT concentration in a microelectrode cells before, during and after applying an electric field: **(A)** 5 mM AOT: NPs form irreversible aggregates. **(B)** 50 mM AOT: NPs form reversible weak concentration gradients. **(C)** 100 mM AOT: NPs form reversible medium-strength concentration gradients. **(D)** 300 mM AOT: NPs form reversible strong concentration gradients.

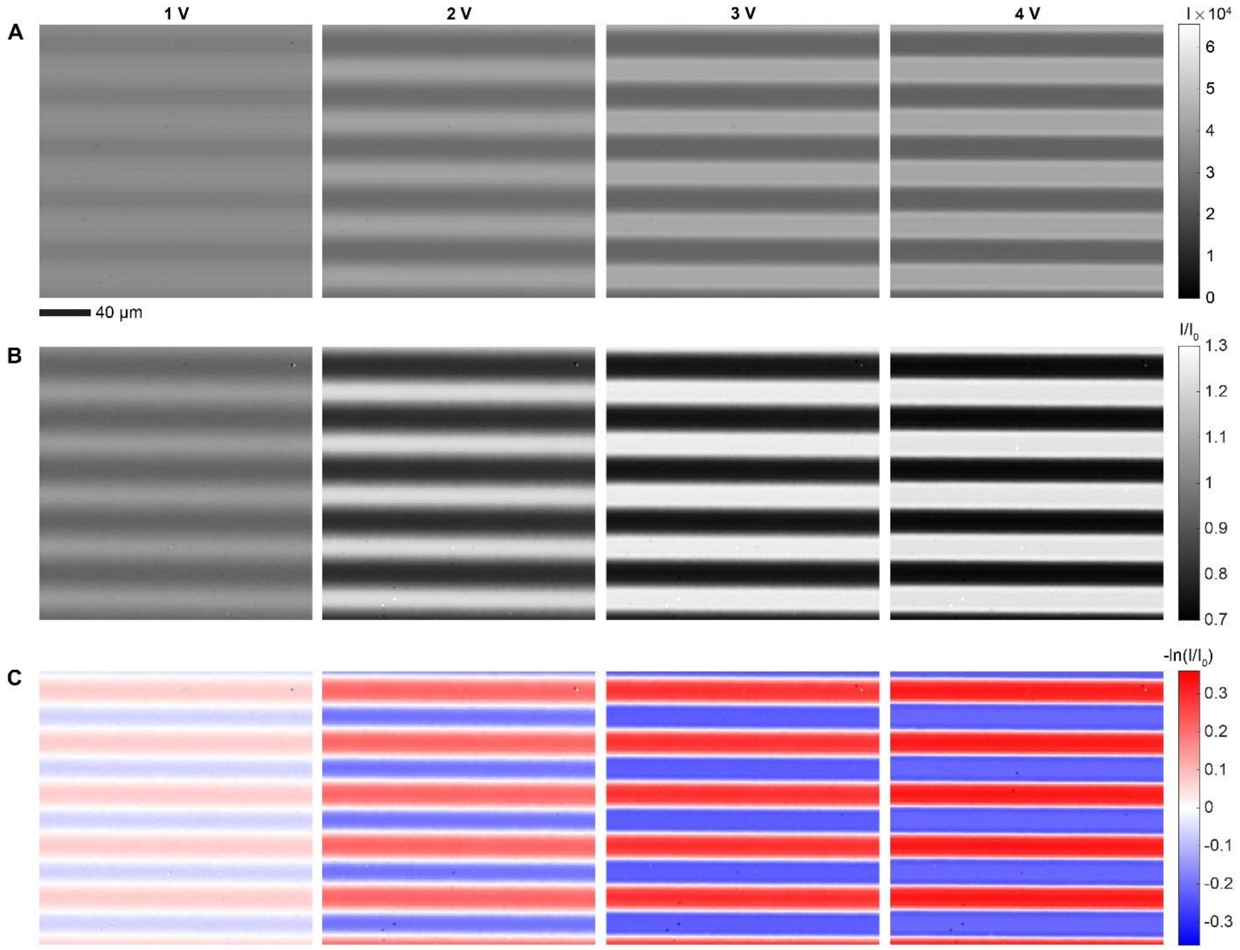

Fig. S5.

Quantification of voltage-controlled magnetism in electroferrofluid. (A) Unprocessed microscopy images of the dissipative steady-states of the electroferrofluid (6 % iron oxide NPs and 150 mM AOT) in a microelectrode cell at four different voltages. Each image is an intensity matrix I with 16-bit intensity values assigned to each pixel. **(B)** Corresponding normalized images calculated by dividing each microscopy image pixel-by-pixel by a reference image I_0 (microscopy image of the equilibrium state, U = 0 V). **(C)** Corresponding images showing $-\ln(I/I_0)$ that is approximately linearly proportional to change in NP concentration and magnetic response.

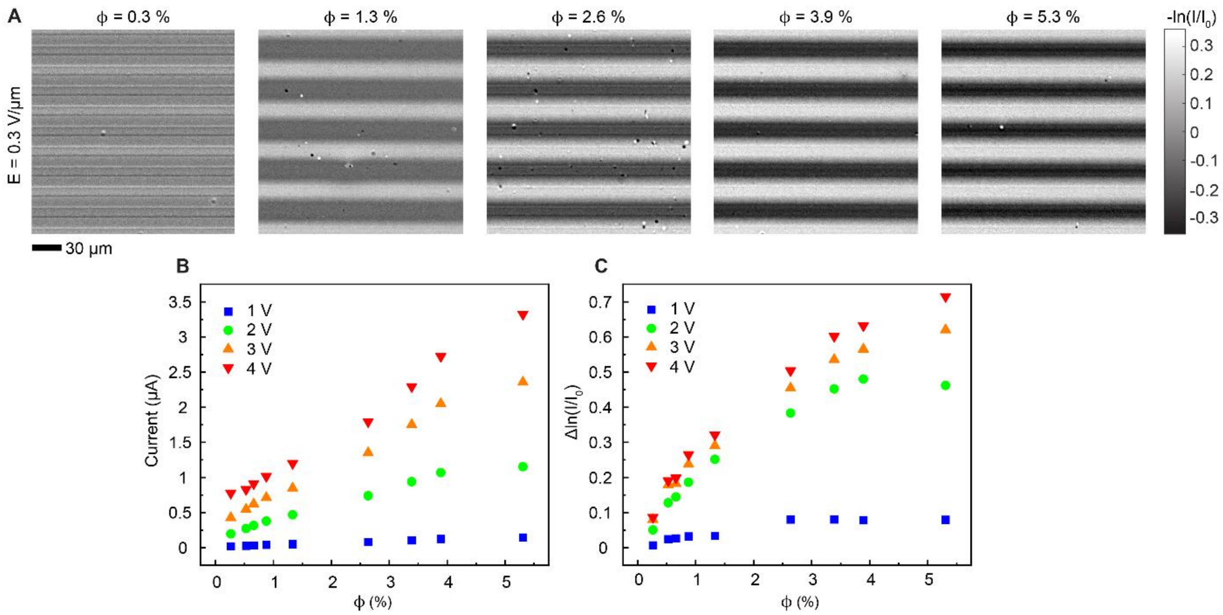

Fig. S6.

Effect of the nanoparticle concentration on the gradient formation in electric field. (A) Images $-\ln(I/I_0)$ of microelectrode cells filled with iron oxide nanoparticles of various concentrations (indicated above each frame) dispersed in dodecane with a constant concentration of AOT (150 mM) at U = 3 V. **(B)** Steady-state current as a function of NP concentration for four different voltages. **(C)** NP concentration gradient amplitude as a function of NP the concentration.

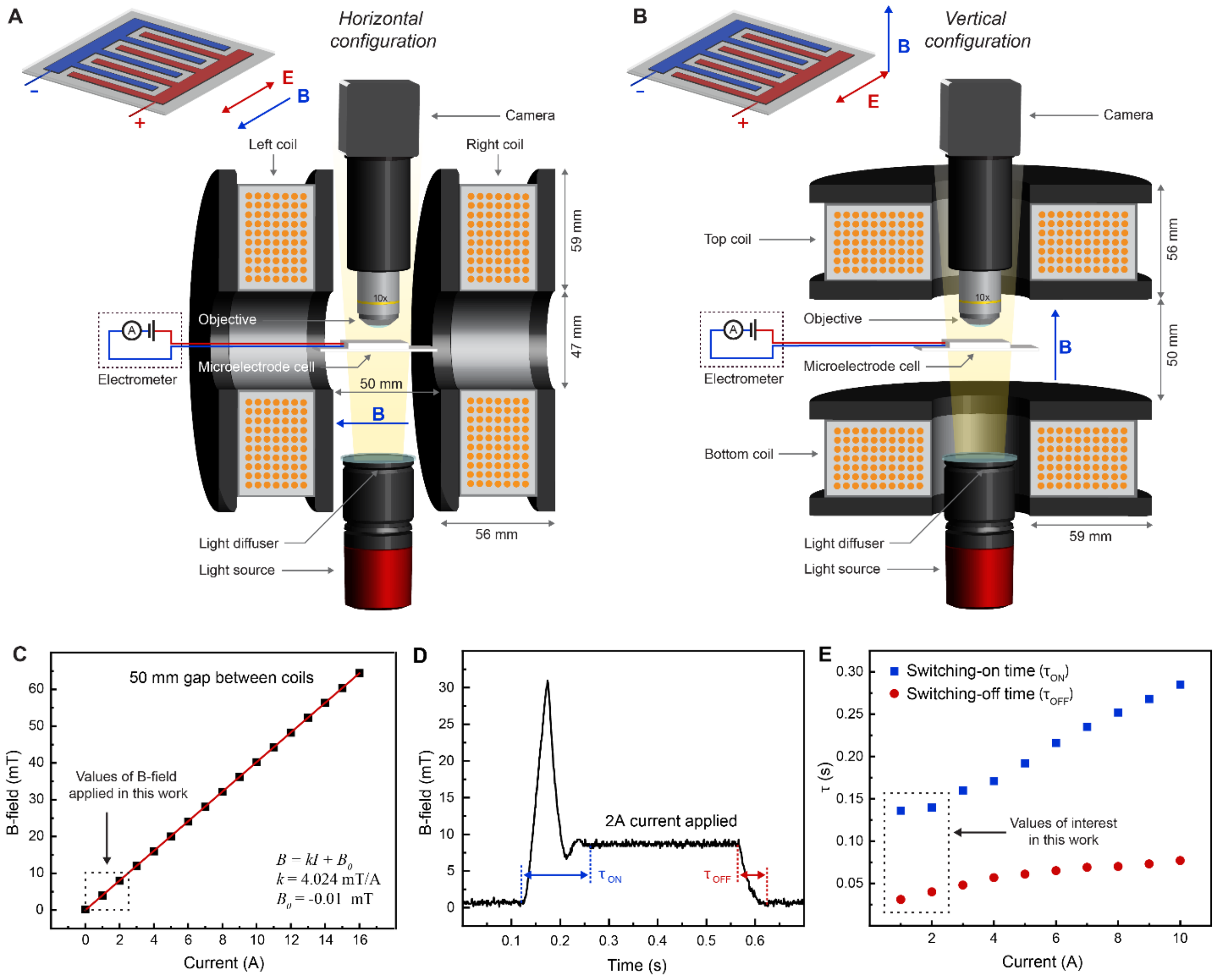

Fig. S7.

Setup for simultaneous microscopic imaging and application of electric and magnetic fields. (A,B) Drawings of the experimental setups used to produce **(A)** horizontal (in-plane) and **(B)** vertical (out-of-plane) magnetic fields. **(C)** Measured steady-state magnetic field as a function of applied current (applies to both in-plane and out-of-plane setups). **(D)** Typical measured magnetic field stabilization behavior over time after turning on the electric current. **(E)** The time required to reach a steady-state magnetic field τ_{ON} and the time required for the magnetic field to be reduced to zero τ_{OFF} upon turning on and off the electric current, respectively.

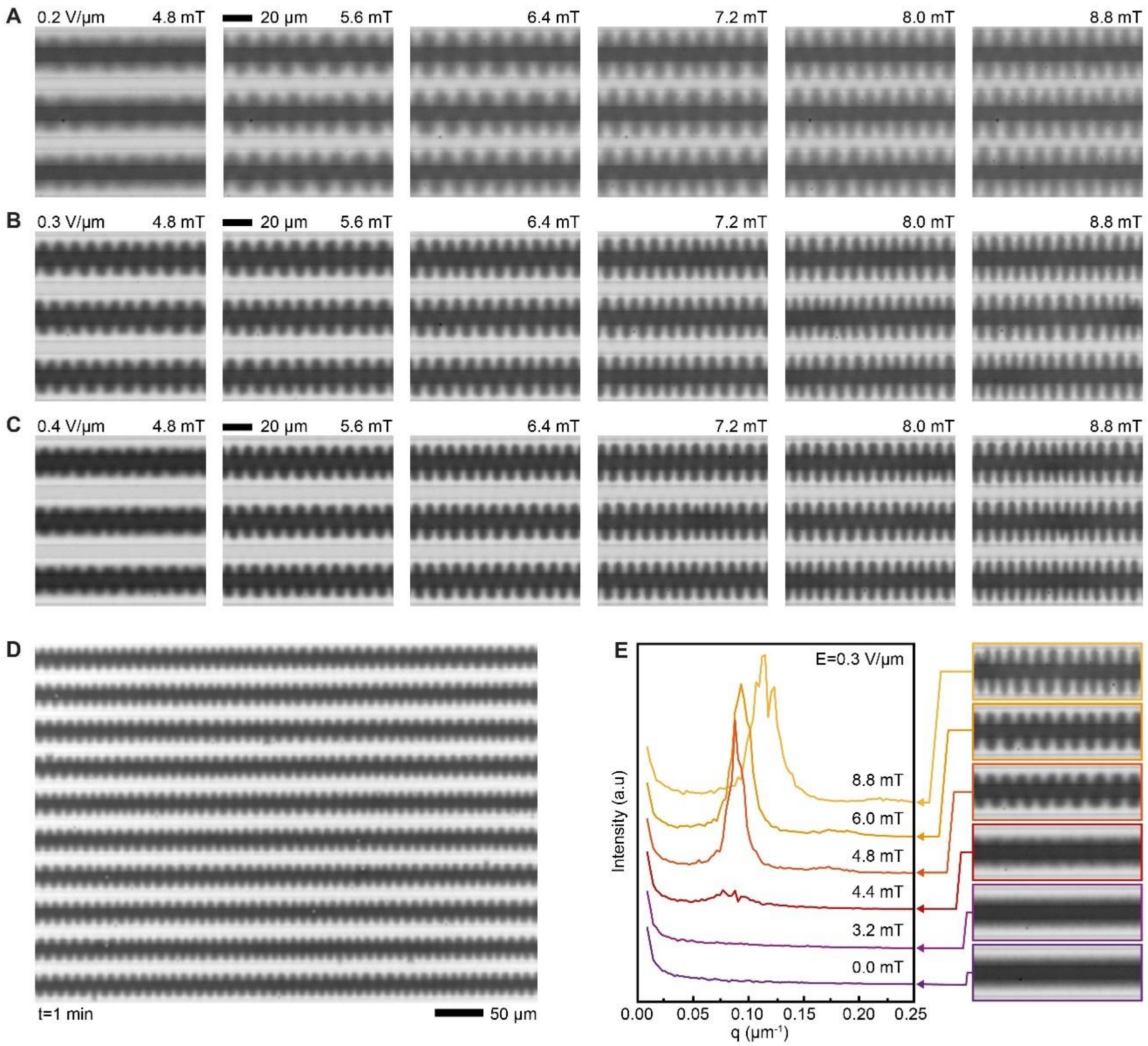

Fig. S8.

Effect of electric and magnetic fields on pattern formation in the out-of-plane magnetic field. (A-C) Microscopy images of the dissipative steady-state of the electroferrofluid (6 % iron oxide NPs and 150 mM AOT) in a microelectrode cell as a function of increasing magnetic field for **(A)** E = 0.2 V/µm, **(B)** E = 0.3 V/µm, and **(C)** E = 0.4 V/µm. **(D)** Low-magnification image of the steady-state pattern (E = 0.3 V/µm, B = 6.4 mT). **(E)** 1D Fourier transformation of the pixel intensity profiles along the electrodes for different magnetic field strengths.

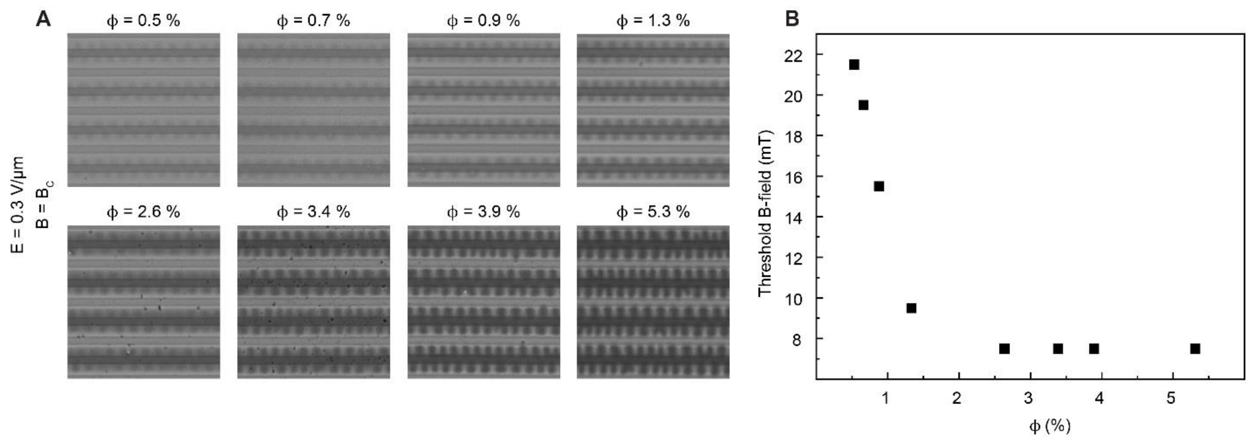

Fig. S9.

Effect of the nanoparticle concentration on the pattern formation in the out-of-plane magnetic field. **(A)** Images of the patterns just above the threshold magnetic field for the onset for pattern formation at $U = 3$ V. **(B)** Threshold magnetic field as a function of nanoparticle volume fraction at $U = 3$ V.

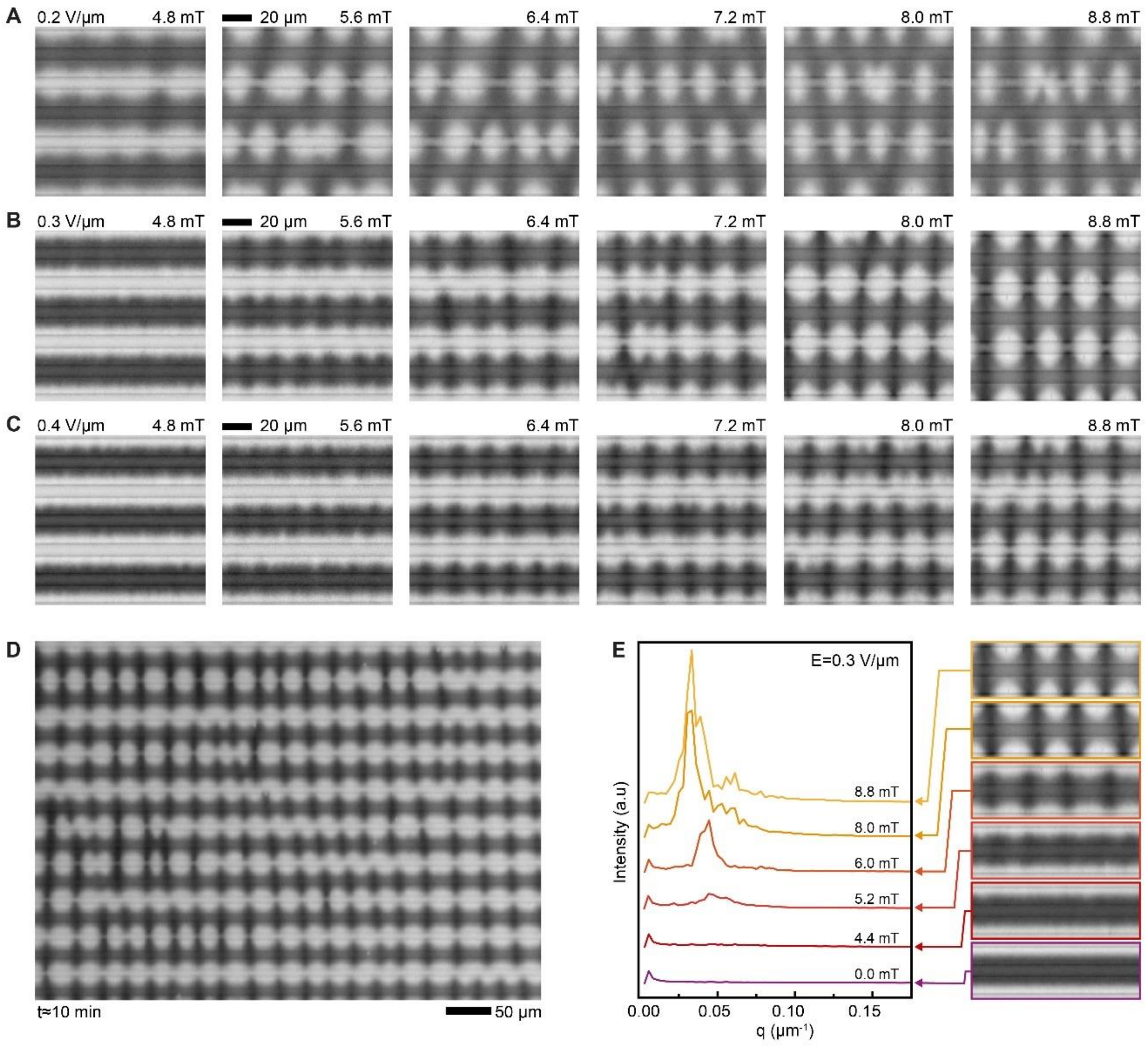

Fig. S10.

Effect of electric and magnetic fields on pattern formation in in-plane magnetic field. (A-C) Microscopy images of the dissipative steady-state of the electroferrofluid (6 % iron oxide NPs and 150 mM AOT) in a microelectrode cell as a function of increasing magnetic field for **(A)**, E = 0.2 V/µm, **(B)** E = 0.3 V/µm, and **(C)** E = 0.4 V/µm. **(D)** Low-magnification image of the steady-state pattern (E = 0.3 V/µm, B = 8 mT). **(E)** 1D Fourier transformations of the pixel intensity profiles along the electrodes for different magnetic field strengths.

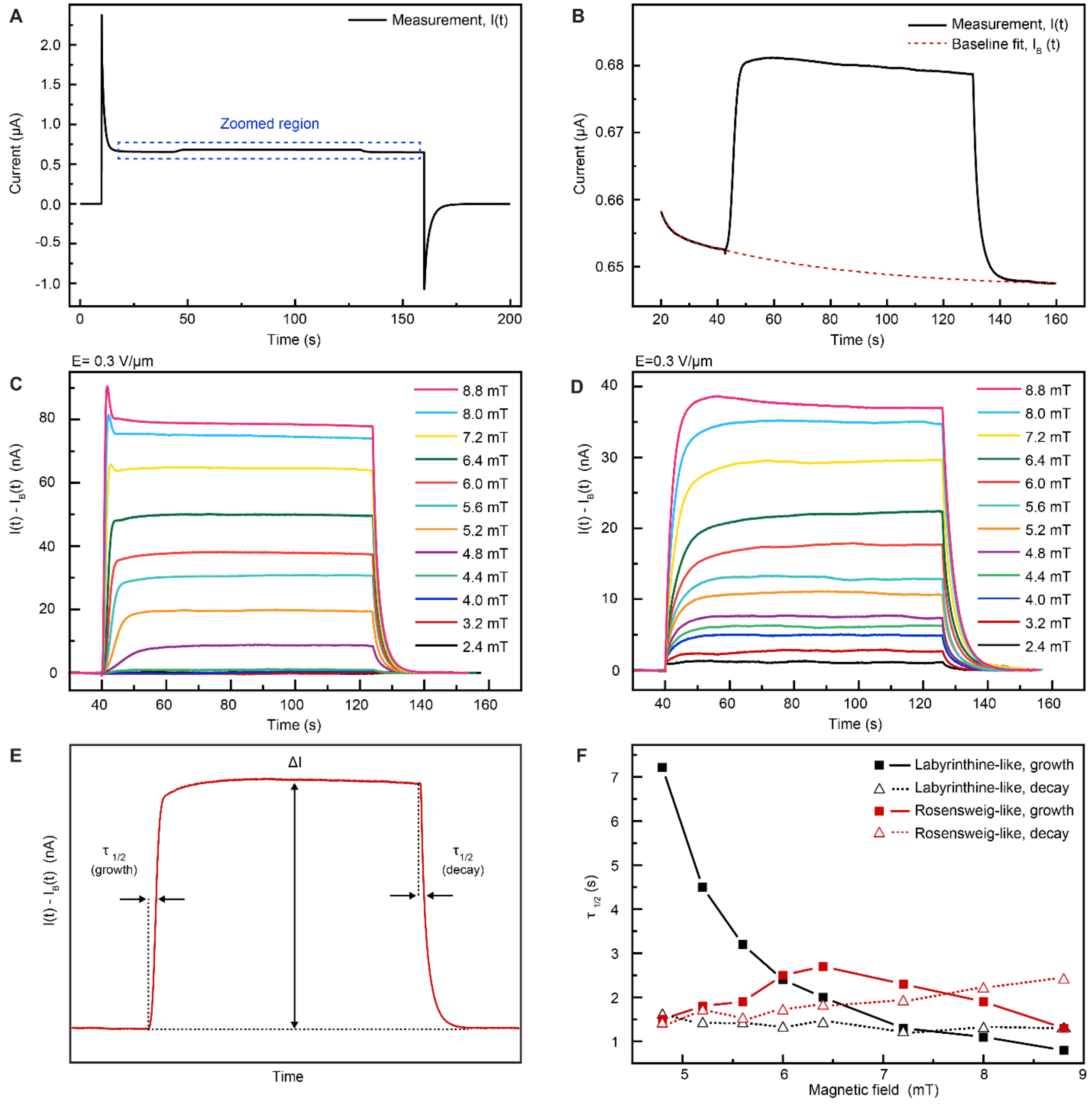

Fig. S11.

Quantification of increase in dissipation during pattern formation. (A) Typical current as a function of time during formation of NP gradient and pattern in electric and magnetic fields, and **(B)** a close-up of the selected region in panel A. **(C)** Change of current for various out-of-plane magnetic field strengths in E = 0.3 V/μm. **(D)** Change of current for various in-plane magnetic field strengths in E = 0.3 V/μm. **(E)** Typical change of current with time constants $\tau_{1/2(growth)}$ and $\tau_{1/2(decay)}$ and steady-state change of current ΔI indicated. **(F)** $\tau_{1/2(growth)}$ and $\tau_{1/2(decay)}$ as a function of magnetic field for labyrinthine-like and Rosensweig-like pattern formation.

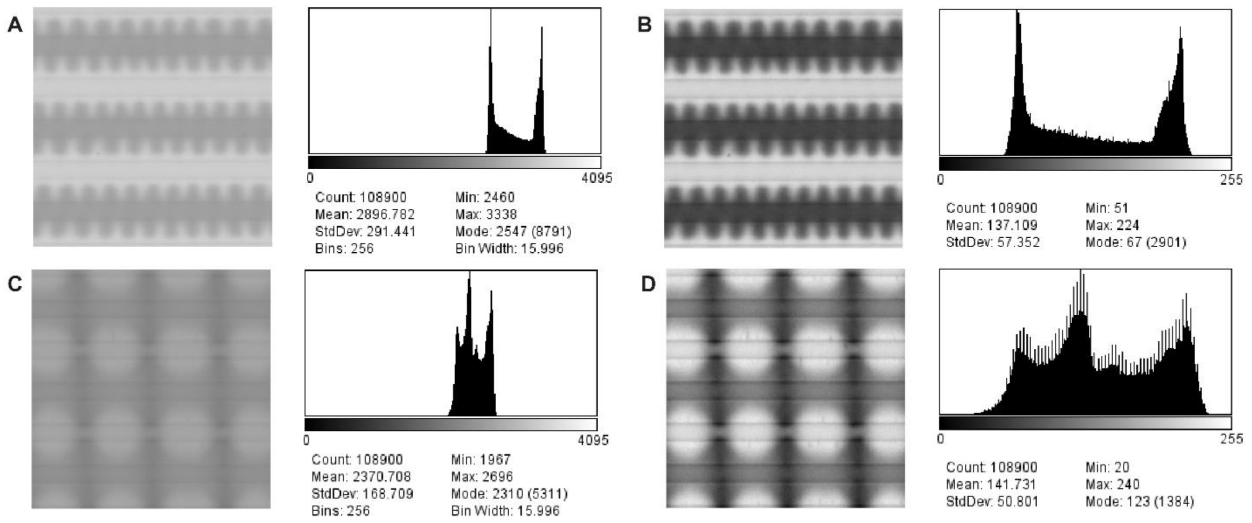

Fig. S12.

Examples of image processing and contrast enhancement. **(A)** Unprocessed 12-bit image of a pattern in an out-of-plane magnetic field as obtained from the camera sensor, and the corresponding intensity histogram. All pixel intensity values are between 2460 and 3338. **(B)** Processed 8-bit image and the corresponding histogram created by linear rescaling of the original image values between 2200 and 3500 to 8-bit range (0 and 255), thus maintaining all pixel values within the range of the new 8-bit image. **(C)** Unprocessed 12-bit image of a pattern in an in-plane magnetic field and the corresponding histogram and **(D)** corresponding processed 8-bit image and histogram.

Movie S1.

Formation of steady-state dissipative NP gradients in an electroferrofluid (6 % iron oxide NPs and 150 mM AOT) driven by electric field. Movieshows a series of microscopy images and plots of electric current and electric field as a function of time in a microelectrode cell filled with an electroferrofluid when an electric field U = 0.3 V/μm is applied for approximately 150 seconds. NPs form steady-state dissipative gradients in electric field that are reversible and disappear once the electric field is turned off.

Movie S2.

Formation irreversible NP aggregates in an (electro)ferrofluid (6 % iron oxide NPs and 10 mM AOT) driven by electric field. Movie shows a series of microscopy images and plots of electric current and electric field as a function of time in a microelectrode cell filled with an (electro)ferrofluid when an electric field U = 0.3 V/μm is applied for approximately 150 seconds. NPs form aggregates in electric field that are irreversible and do not disappear once the electric field is turned off.

Movie S3.

Pattern formation in electroferrofluid (6 % iron oxide NPs and 150 mM AOT) in 6 mT out-of-plane magnetic field. Movie shows a series of microscopy images and plots of electric current, electric field and magnetic field as a function of time in a microelectrode cell filled with an electroferrofluid when an electric field U = 0.3 V/μm is applied and followed by application of magnetic field (6 mT, out-of-plane). NPs first form steady-state dissipative gradients in electric field and further a labyrinthine-like pattern in magnetic field. Both pattern and gradient are reversible once magnetic and electric fields are turned off, respectively.

Movie S4.

Pattern formation in electroferrofluid (6 % iron oxide NPs and 150 mM AOT) in increasing out-of-plane magnetic field. Movie shows six series of microscopy images of a microelectrode cell filled with an electroferrofluid when an electric field U = 0.3 V/μm is applied and followed by application of six different magnetic fields (from 3.2 to 8.8 mT, out-of-plane). In all cases, NPs first form steady-state dissipative gradients in electric field and further labyrinthine-like patterns in magnetic field with decreasing pattern periodicity with increasing magnetic field strength.

Movie S5.

Pattern formation in electroferrofluid (6 % iron oxide NPs and 150 mM AOT) in 8 mT in-plane magnetic field. Movie shows a series of microscopy images and plots of electric current, electric field and magnetic field as a function of time in a microelectrode cell filled with an electroferrofluid when an electric field U = 0.3 V/μm is applied and followed by application of magnetic field (8 mT, in-plane). NPs first form steady-state dissipative gradients in electric field

and further a Rosensweig-like pattern in magnetic field. Both pattern and gradient are reversible once magnetic and electric fields are turned off, respectively.

Movie S6.

Pattern formation in electroferrofluid (6 % iron oxide NPs and 150 mM AOT) in increasing in-plane magnetic field. Movie shows six series of microscopy images of a microelectrode cell filled with an electroferrofluid (150 mM AOT in dodecane) when an electric field U = 0.3 V/μm is applied and followed by application of six different magnetic fields (from 4.4 to 8.8 mT, in plane). In all cases, NPs first form steady-state dissipative gradients in electric field and further Rosensweig-like patterns in magnetic field with increasing amplitude with increasing magnetic field strength.
